# Perceived gender and political persuasion: a social media field experiment during the 2020 US Democratic presidential primary election

**DOI:** 10.1038/s41598-023-39359-0

**Published:** 2023-08-28

**Authors:** Aidan Combs, Graham Tierney, Fatima Alqabandi, Devin Cornell, Gabriel Varela, Andrés Castro Araújo, Lisa P. Argyle, Christopher A. Bail, Alexander Volfovsky

**Affiliations:** 1 Department of Sociology, Duke University, Durham, USA; 2 Department of Statistics, Duke University, Durham, USA; 3 Department of Political Science, Brigham Young University, Provo, USA

**Keywords:** Psychology, Human behaviour

## Abstract

Women have less influence than men in a variety of settings. Does this result from stereotypes that depict women as less capable, or biased interpretations of gender differences in behavior? We present a field experiment that—unbeknownst to the participants—randomized the gender of avatars assigned to Democrats using a social media platform we created to facilitate discussion about the 2020 Primary Election. We find that misrepresenting a man as a woman undermines his influence, but misrepresenting a woman as a man does not increase hers. We demonstrate that men’s higher resistance to being influenced—and gendered word use patterns—both contribute to this outcome. These findings challenge prevailing wisdom that women simply need to behave more like men to overcome gender discrimination and suggest that narrowing the gap will require simultaneous attention to the behavior of people who identify as women and as men.

## Introduction

Women have less influence than men in a variety of decision-making settings such as business^1–3^, education^4,5^, academia^6,7^, politics^8–10^, and interpersonal conversations more broadly^11,12^. Even when women report feeling satisfied with a discussion or negotiation, studies reveal they have less influence on the decisions made or the views of fellow group members than men^8,13,14^. Pervasive discounting of women’s expertise, competence, or capability reduces women’s confidence^15^, influence^16^, and aspirations^17^, and presents a barrier for women’s career advancement into high-level leadership roles in STEM^17^, business^18,19^, politics^20^, sports^21^, medicine^22^, and many other areas. Yet women bring distinctive experiences, priorities, and approaches to policy-making processes^8,14,23,24^, and their absence leads to unrepresentative outcomes and lower trust in decision-making institutions^25,26^. Improving women’s credibility and influence is thus a critical challenge to break through the glass ceilings that undermine women’s representation in many fields.

In this article, we ask: What causes women’s lower influence in discussions about politics, and how might it be improved? Previous research indicates gender gaps emerge in social contexts where people already have expectations of gender norms for a particular domain^27–31^. Incongruities between gender role expectations (what people expect men and women to do or say in a given setting) and performance of gender (what men and women actually do or say) both contribute to gender inequality^30^. While there is ample evidence for both dynamics functioning independently, the complex interactions between expectations and performance remain poorly understood in real-life settings. We contribute to this body of work with an innovative experimental design that allows us to simultaneously examine both explanations within the heavily gendered discourse surrounding the 2020 Democratic Primary election in the United States in a social media-like environment.

Studies that explain the gender gap in political influence as the result of differential treatment of men and women speakers emphasize the role of stereotypes—often independent of differences in the actual behavior of men and women^32^. The notion that women are less competent than men in politics persists even a century after women’s suffrage^33^, though such differences may be contingent on partisanship^34,35^. The incongruity between gender and political roles is often described as a conflict between agentic traits (e.g. decisiveness, assertiveness, competence) that are typically associated with both political leadership and masculinity and communal traits (e.g. friendliness, cooperation, helpfulness) that are typically associated with cooperative teamwork and femininity^36–40^. Even when accounting for objectively measured levels of political knowledge^41,42^, people engaged in discussions about politics view women as less competent and knowledgeable^43^. Women’s contributions can be discounted through subtle behaviors such as interrupting women when they speak^8,44,45^, not giving women credit for their ideas or equal work^46^, or *mansplaining* in online discourse^47^. While women may not be more likely than men to be targets of incivility online^48,49^, the experience of online harassment has a depressive effect on women’s future participation in online discourse^48,50^. Over time, these processes systematically undervalue the contributions of women^51^ and make it more difficult for women to succeed in politics and other leadership roles^30^.

Gender performance—or the practices and habits of femininity and masculinity that women and men use to communicate gender—may also contribute to women’s lower influence, because male-typed behaviors are more highly valued in some settings than female-typed behaviors. Studies of gender performance emphasize differences in word choice, tone, or behaviors^52–57^, particularly if these speech patterns are seen as less authoritative. For example, in business settings, feminine-stereotyped behaviors in venture capital pitches result in less investor preference, likely because the evaluators interpret them as signals of lower competence^58,59^. Highly qualified women are also less likely than men with comparable skills to be contributors to online information repositories, such as Wikipedia^60^. In politics, women have lower levels of self-confidence^61^, are more likely to avoid conversations that might lead to confrontation^62,63^, and are less likely to try to influence the votes of others^9^ or correct their views^64^. Specifically in online environments, women are less likely to comment on news sites or post about politics^49,64,65^. This limits the scope of women’s influence in political disagreements^66^, their participation in politics^67^, and their ambitions to seek office^68,69^.

While these are not competing explanations, the majority of the research just described attempts to isolate and evaluate a single explanatory theory, often using research designs that hold core features of the other theory constant. This leads to an incomplete understanding of the complexity of gender dynamics in interpersonal interactions—particularly in online spaces that allow people greater freedom to control gendered cues in their self-presentation—and the promotion of solutions based on changing other actors’ gender biases^32^, or encouraging women to change their behavior or “lean in”^70^, but rarely both. We contribute to this literature with a large-scale field experiment on a custom-built social media platform that allows us to simultaneously evaluate the relative weight of each explanation.

In this experiment, we randomly paired two people who identified as Democrats to have an online, text-based conversation about the 2020 Presidential Primary Election in the United States. In the control condition, a woman and a man had a conversation together, and each participant was represented by a gendered avatar that was visible only to their conversation partner. In some treatment conversations, we manipulated gender perceptions by randomly varying whether these avatars were consistent or inconsistent with the partner’s self-reported gender identity. In another set of conversations, we paired respondents with a conversation partner of their same gender with a correctly-gendered avatar. This design allows us to examine how perceptions of gender interact with gender performance as both unfold over the course of a conversation.

In particular, if women who are mislabeled as men gain relative influence in the conversation, this would be evidence that gendered expectations are a main cause of women’s lower influence in interpersonal settings. Under this explanation, we would also expect little difference in the gendered content of the language used by men and women. By contrast, if women who are mislabeled as men experience no change in their relative influence compared to women who are not mislabeled, this would be evidence that sexism in the response to how women perform their gender is a primary cause of women’s lower influence. This theory also predicts identifiable and relatively static differences in the gendered language used by men and women, regardless of the avatar assigned. However, if both explanations are simultaneously occurring, then role incongruity might lead to a decrease in influence for both men and women when their gender is misrepresented^30^, and dynamics of the unfolding conversation may cause language patterns to shift and send mixed signals about gender^55,71^.

## Experimental design

We conducted a field experiment during the 2020 U.S. Democratic presidential primary election on a text-based social media platform designed for academic research,(see Fig. 6). Our study was approved by an Institutional Review Board. The chat platform allowed for people to engage in an anonymous, real-time political conversation about the Democratic primary candidate best poised to beat Donald Trump in the general election. Prior to the conversation, one-third of respondents selected Joe Biden as their top candidate and one-third selected Bernie Sanders, with no gender difference in preference for or rating of those candidates. Elizabeth Warren, the third-ranked candidate, was more preferred by women (16%) than men (8%), although the thermometer ratings of Warren did not differ by gender.

**Figure 1:**
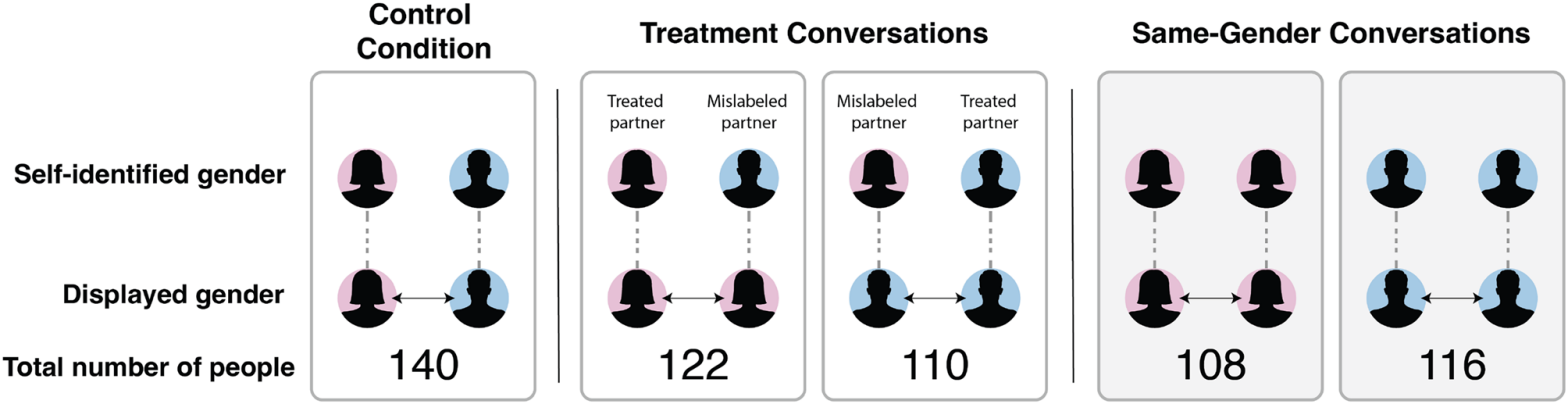
Research design. In the control condition, one man talks to one woman, and both discussants are represented by an avatar associated with their self-reported gender. The treatment conditions also pair one man and one woman, but the treated discussion partner views an avatar that does not match the self-identified gender of their mislabeled discussion partner. The same-gender conditions pair men to converse with another man and women to converse with another woman, and both discussants are represented by an avatar associated with their self-reported gender. Note that contrasting the same-gender and any cross-gender conversations does not identify the causal effect of gender because only partners were randomized, not partner gender (holding other partner characteristics fixed). Men and women differ on many unobserved characteristics, so changing someone’s discussion partner from a man to a woman changes more than just the partner’s gender. See Supplementary Appendix Sect. 11.4 for more details.

In the conversation, gender presentation is manipulated through random assignment of a male or female avatar for some respondents. The text-based conversation means that gendered patterns develop through an ongoing social interaction, but that gender can only be communicated through the display avatar and the text of messages sent, with no interference from physical, visual, or vocal cues.

Participants were randomly assigned to one treatment or control conversation, and the experimental effects are estimated by comparing the between-subjects differences in averages using *t*-tests. In the control condition, one self-identified male respondent had a conversation with one self-identified female respondent, and each respondent viewed an avatar for their partner that accurately reflected their gender identity. In two experimental conditions, the conversations remain cross-gender, but one of the partners was randomly selected to see an avatar for their discussion partner that did not match the partner’s self-reported gender identity. In two additional conditions, subjects were matched with a partner of their same gender, and the avatars correctly portrayed their gender. Figure 1 illustrates the research design. All respondents were debriefed about the avatars used to depict them after the study was concluded. We note here that in our comparisons between same-gender and cross-gender conversations it is not possible to distinguish between the effects of having a discussion partner of a different gender and other potential gendered mechanisms in the conversation. Nevertheless, we report these comparisons as they provide suggestive evidence for potential mechanisms for the experimental results.

## Results

We report our results using four types of outcomes. First, we compare the gender gap in the level of influence of each partner in a cross-gender conversation. The gap is evaluated using three metrics, and their composite index: (1) the partner’s subjective survey report of the subject’s influence on their attitudes, (2) the pre-post change in the partner’s thermometer rating of the candidate most preferred by the subject in the pre-survey, (3) the pre-post change in the partner’s ranking of the candidate most preferred by the subject in the pre-survey. The index provides an indication of how much influence a person has in a conversation relative to the influence of their partner, and the average difference between partners provides a metric of the gender gap. Our expectations for the value of this metric for each competing theory are provided in Table 1.

**Table 1:** Theoretical expectations of influence gap.

Hypothesized source of gap	Theoretically expected effect	Sign of gap
*Control condition: men talk to women*		
No intervention; status quo	Men will have more influence	+
*Treatment conditions*		
**Women talk to men mislabeled as women**		
Gendered stereotypes	Equivalent to a same-gender conversation with no expected average difference	0
Gendered performance	No change to man’s influence because nothing about the man’s behavior will have changed	+
Stereotypes and performance	A perceived woman talks in ways more characteristic of men, which produces a negative response due to role incongruity	–
**Men talk to women mislabeled as men**		
Gendered stereotypes	Equivalent to a same-gender conversation with no expected average difference	0
Gendered performance	No change to woman’s influence because nothing about the woman’s behavior will have changed	+
Stereotypes and performance	A perceived man talks in ways more characteristic of women, which produces a negative response due to role incongruity	more +
*Additional reference: same gender conversations*		
No intervention; status quo	Equal influence on average	0

Second, we look at a conversation-level metric of convergence in the thermometer ratings of the full set of candidates, which shows how attitudes converge or diverge at the conversation level and beyond just the evaluations of the top-ranked candidate. Third, we examine the influence metrics (the same set as the gender gap analysis) at the individual level, to more closely examine the dynamics of who has influence and whose influence is changing in response to the experimental intervention. Finally, we use a dictionary-based evaluation of gendered language to compare the gendered language used by men and women in each type of conversation.

### Influence gap

**Figure 2:**
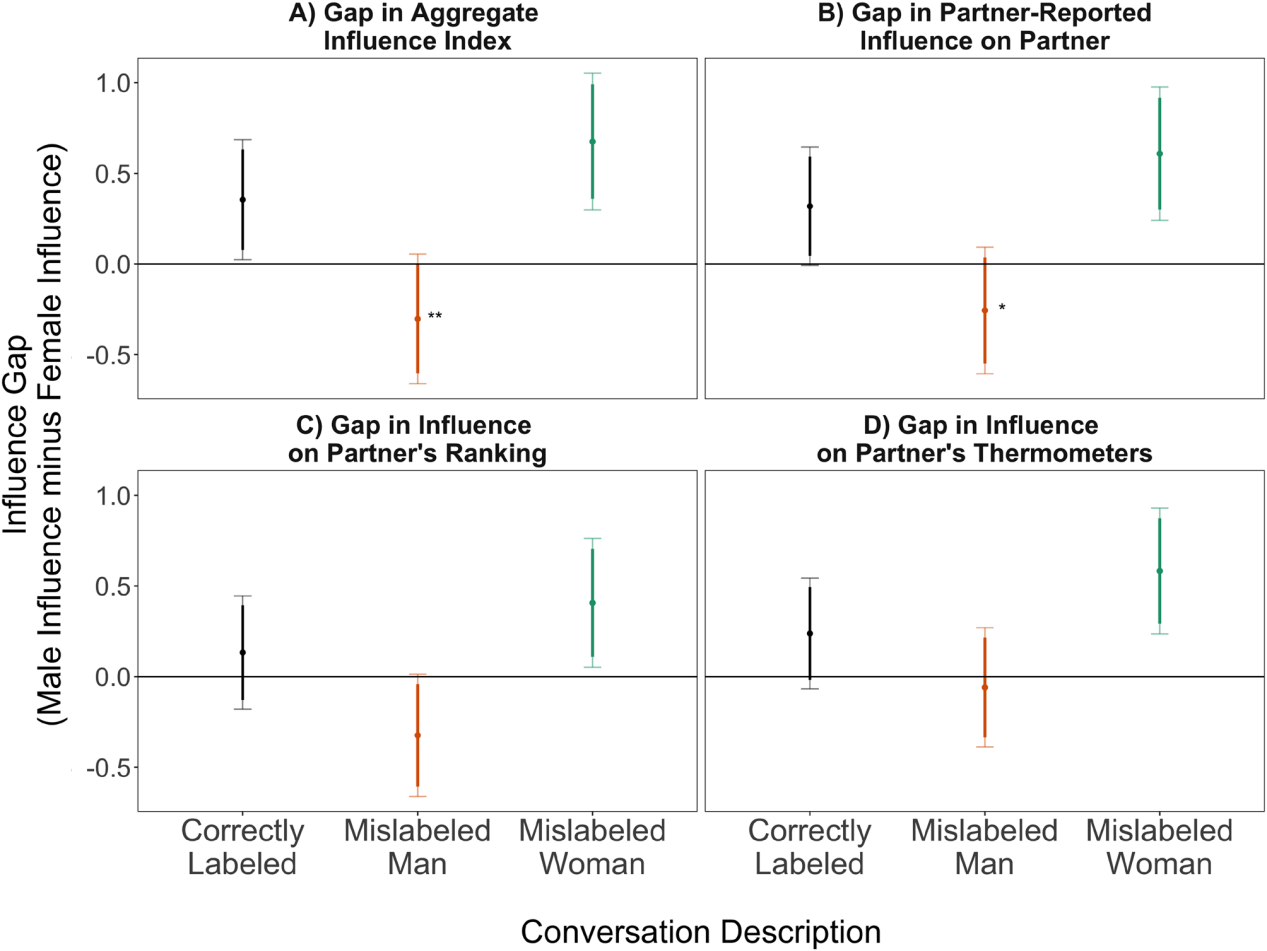
Effects of gender mislabeling on the influence gap in cross-gender conversations about the 2020 Democratic Primary Election. The influence gap measures the difference between the influence of the man and woman on the given measure. Positive values indicate that the man is more influential. Dots are point estimates of the gap with 90% and 95% confidence intervals. Stars indicate significant differences between mislabeled conversations (orange for mislabeled men or green for mislabeled women) and correctly labeled conversations (black) using two-tailed t-tests. One star indicates significance at the 5% level, two indicates significance at the 1% level. In correctly labeled conversations, men are more influential on all metrics—and significantly so for the aggregate index ($p=0.036$, standard error 0.168, *t*-statistic 2.11 with 147 degrees of freedom). The orange and green bars show the influence gap in discussions where one partner was mislabeled. Mislabeling men as women reverses the gap such that women are on average the more influential partner in those conversations ($p=0.009$, standard error 0.247, *t*-statistic − 2.664 with 147 degrees of freedom). Mislabeling women as men increases men’s relative influence, widening the gap, though these effects are not statistically significant. The figure shows unadjusted means because the levels are interpretable and meaningful, consequently statistical significance is reported with *t*-tests that do not adjust for demographic covariates. Our randomization produced treatment and control groups balanced on these covariates, and statistical significance is identical with adjusting for them. See Table S13 for full statistical results and Table S14 for results with demographic covariates.

Figure 2 presents the average difference in men’s and women’s influence in cross-gender conversations for three metrics and the composite (in Panel A). Positive values indicate that the man in the conversation had more influence, negative values indicate that the woman had more influence, and a score of zero would indicate equal influence from both partners. All panels display the conditional mean value along with 90% and 95% confidence intervals. Asterisks indicate significant differences relative to the control condition using two-tailed tests. As expected, men in the control conversations (black lines) are more influential than women—demonstrated by the positive mean values on all metrics. In same-gender conversations, the gender gap metric is by definition always zero, so those conditions will only be discussed in the individual-level metrics section.

The results for both treatment conditions are inconsistent with either the gender stereotypes or gender performance theories alone. Rather, when men are mislabeled as women (orange lines), men’s influence is reduced such that the influence gap changes direction, meaning that—relative to their male partners—women are on average the more influential partner when their male partners are misrepresented as women. Women’s influence likewise does not improve when they are misrepresented as men—if anything, women lose relative influence in those conversations compared to the control, but these differences are not statistically significant.

These results indicate that, regardless of one’s actual gender, mislabeling someone’s gender reduces (or at least does nothing to improve) their level of political influence relative to their partner. This outcome is consistent with sociological explanations that predict negative effects when people’s actual behaviors contradict stereotyped expectations for how they should behave in a particular setting^27,30^, which is a result of both gender stereotypes and gender performance having simultaneous influence.

### Attitude convergence

To further explore the consequences of the mislabeling intervention for the overall trajectory of conversations, we examine a conversation-level metric of convergence in thermometer rankings across all candidates. We compare the average gap between the subject’s rating of each candidate and their partner’s rating of the same candidate, before and after the conversation. This metric has the advantage of looking at changes in more than just one subject’s top-rated candidate, and it allows for reciprocal influence across the full range of candidates. Same-gender conversations are excluded from this analysis because the initial level of agreement on candidates between conversation partners is different than for cross-gender conversations due to gendered differences in candidate preferences (see the Supplemental Appendix). We do see that people in the control condition begin their conversations with a larger thermometer gap than people in conversations where one partner is mislabeled. This could be due to differential attrition across the three conditions, though we believe this issue is unlikely to impact the actual findings reported here. See the Supplemental Appendix for analysis and discussion of this issue.

**Figure 3:**
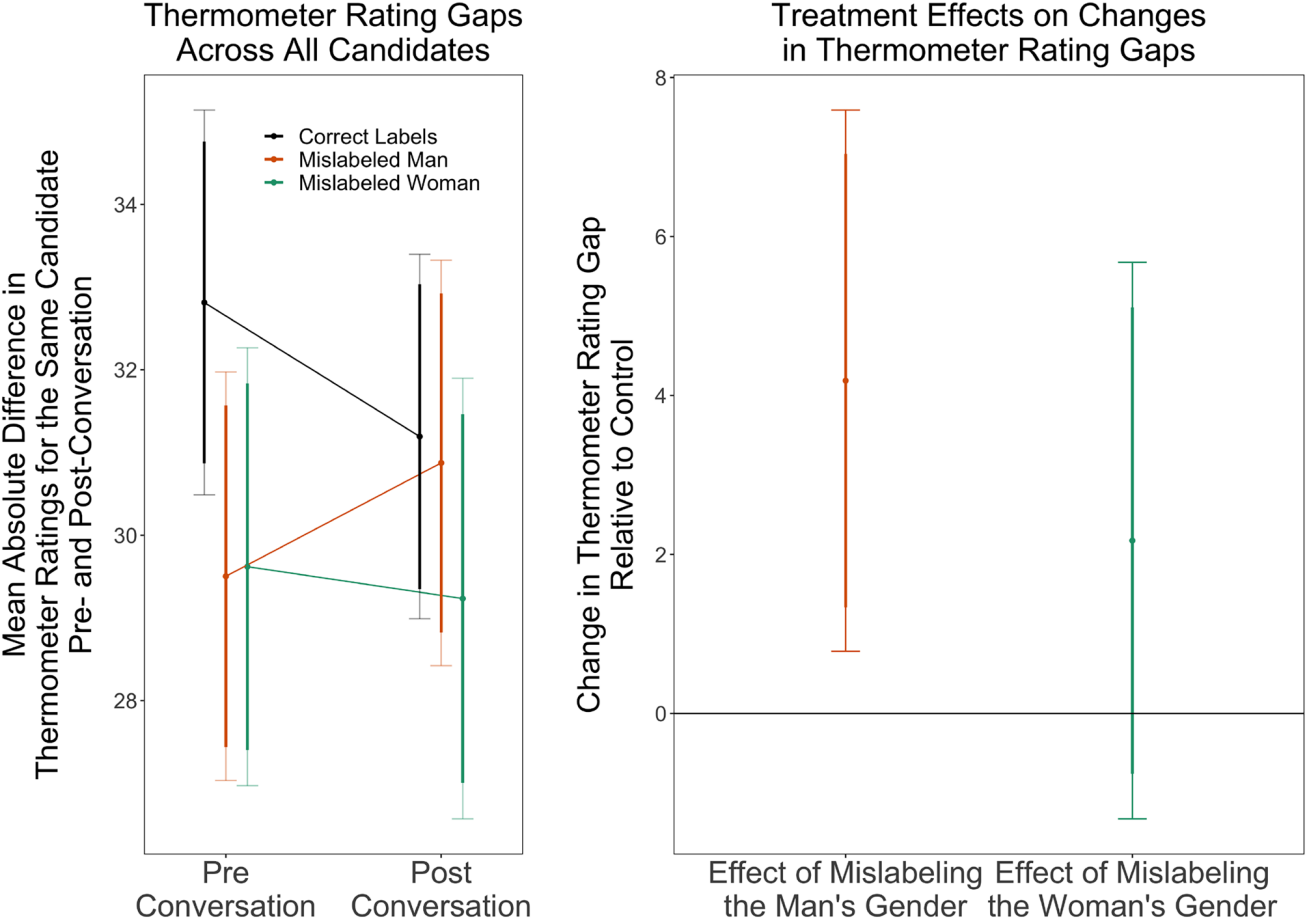
Mislabeling effects on attitude convergence. The left panel shows the magnitude of disagreements in feelings towards candidates pre- and post-conversation by treatment condition and the right panel explicitly shows treatment effects on the within-conversation changes in feelings towards candidates. This visualization depicts the absolute difference in thermometer rating for the same candidate across conversation partners within either the pre- or post-conversation survey. Conversations where the man is mislabeled exhibit increases in disagreement that are significantly larger (shown in the right panel, two-tailed $p=0.016$, standard error 1.73, *t*-statistic 2.43 with 182 degrees of freedom) than the decreases in disagreement in correctly labeled conversations. Mislabeling the woman increases disagreement relative to control but not at statistically significant levels. 95% and 90% confidence intervals are shown. The figure shows unadjusted means because the levels are interpretable and meaningful, consequently statistical significance is reported with *t*-tests that do not adjust for demographic covariates. Our randomization produced treatment and control groups balanced on these covariates, and statistical significance is identical with adjusting for them. See Table S17 for full statistical results and results adding demographic covariates.

The left panel of Fig. 3 presents the average gap across all thermometer ratings for each conversation type. While correctly labeled cross-gender conversations exhibit a substantial reduction in the average gap in thermometer ratings from pre- to post-treatment measurement, the gap remains constant for conversations in which women are mislabeled as men. Conversations in which men are mislabeled as women actually see an increase in the thermometer rating gap, meaning attitudes diverged as a result of the conversation. This divergence is significantly different (p < 0.05, two-tailed test) from behavior in correctly-labeled, cross-gender conversations, as shown in the right-hand panel. One interpretation of the influence gap results is that just changing how a female conversation partner perceives or reacts to the gender of their male discussion partner might be a way to improve gender equity in cross-gender political conversations. However, although women are more influential on average in conditions where men have been mislabeled (see Fig. 2), the increased divergence of overall attitudes suggests that merely changing how men are perceived in a conversation is unlikely to move conversations towards more consensus outcomes or gender-balanced influence.

### Individual-level influence metrics

**Figure 4:**
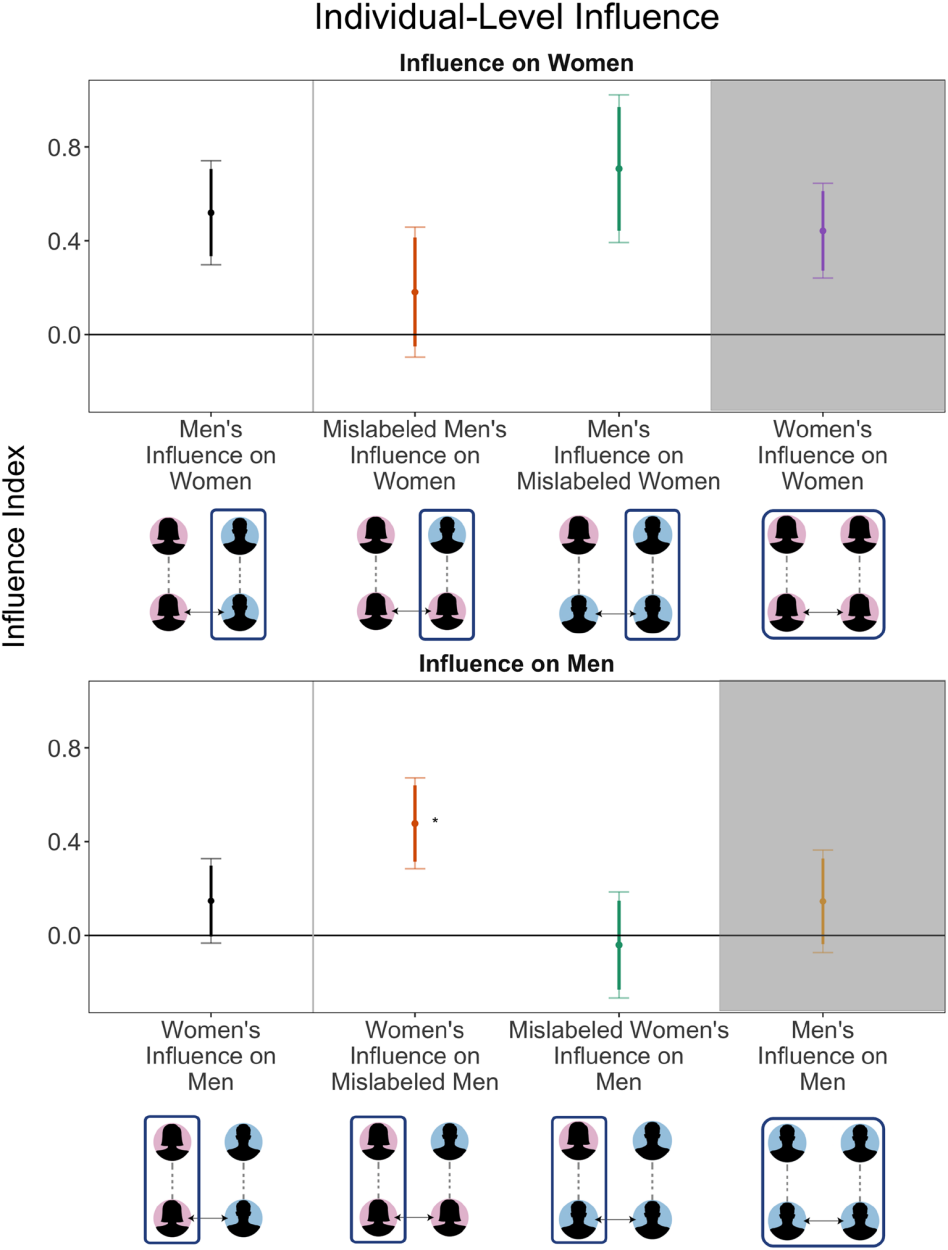
Average influence on partners by partner’s gender and treatment condition. Point estimates and error bars indicate average level of influence exerted by the subject. Influence is measured with the aggregate influence index (panel A in Fig. 2); effects are the same direction for all component measures. Mislabeling men’s gender significantly increases women’s influence on men ($p=0.014$, standard error 0.134, *t*-statistic 2.46 with 277 degrees of freedom), relative to women’s influence in a correctly-labeled conversation. Thicker lines are 90% confidence intervals, the error bars extend to make 95% intervals. Stars indicate statistically significant (p < 0.05) differences between the indicated influence and influence in the control condition, correctly-labeled conversations between men and women. All statistical tests are two-tailed. Standard errors are clustered at the conversation level. The same-gender conversations are shaded grey to indicate that they do not identify causal effects of gender. The figure shows unadjusted means because the levels are interpretable and meaningful, consequently statistical significance is reported with *t*-tests that do not adjust for demographic covariates. Our randomization produced treatment and control groups balanced on these covariates, and statistical significance is identical with adjusting for them. See Table S18 for full statistical results and Table S19 for results with demographic covariates.

Because a conversation is a dynamic interaction between two people, the change in the influence gap metrics might come from two mechanisms—a change in the persuasiveness of one partner, or a change in the other partner’s propensity to be influenced. In order to distinguish these two mechanisms, we evaluate the treatment effects of mislabeling on both the influence exerted by and the propensity to be influenced of each discussion partner separately. Fig. 4 shows the mean value of the aggregate influence index (see Panel A of Fig. 2), for respondents by gender and treatment condition.

The top panel represents the level of persuasiveness of respondents when the person they are influencing is a woman, whereas the bottom panel represents influence on a male partner. The difference between the top and bottom panels for cross-gender conversations is the influence gap measure presented in panel A of Fig. 2. There is a notable difference between the typical level that men and women are influenced. Men (bottom panel) are less likely than women to be influenced in every condition—and indeed, show no significant signs of having been influenced—except for when they themselves have been mislabeled as women. Women (top panel) by contrast, are themselves influenced by their partner in every condition except when their male partner is mislabeled as female.

Contrary to expectations, women do not have less influence in the conversation than men *when the gender of the person they are trying to persuade is held constant*. In other words, when women are the target, men and women are equally persuasive (first and last estimates in the top panel), and when men are the target, men and women are equally non-persuasive (first and last estimates in the bottom panel). This suggests that one reason why women have less influence in mixed-gender settings is because their partners are typically men who are less amenable to being influenced.

Figure 4 provides a nuanced and unexpected account of how gender perceptions affect the dynamics of influence in interpersonal conversation. In particular, when women are talking to a man who has been mislabeled as a woman, they have an unexpectedly high amount of influence on their partner. Although their conversation partner is still a man, when women think they are talking to another woman, they are able to exert more influence on their conversation partner.

While our initial emphasis was on increasing women’s influence by changing how they themselves are perceived by their male partners, what we discover is that changing how women perceive (and, therefore, treat) their male partner had a bigger impact. This finding is particularly notable because it suggests that men are not inherently or immutably less persuadable than women—they are only less persuadable when their partners recognize them as men. In the experimental condition where women do not realize they are talking to a man, women are able to exert more influence on their partner. Put another way, men’s attitudes are more malleable when they are not treated like men, which suggests that the assumptions and behavioral decisions that discussion partners make when interacting with men reinforce the influence gap, possibly even more than the assumptions and behavioral decisions that people make when interacting with women.

The individual-level analysis provides evidence that both gendered behavior and gendered perceptions interact in complex ways within a dynamic conversation. Although only one partner is treated (views the mis-assigned gendered avatar for their partner), the treatment has effects on both parties in the conversation. Because their perceptions are not directly manipulated, the only way effects on the mislabeled partner are possible is if their partner changes their language or conversational style in response to the gendered avatar of their partner, and they respond in kind. We examine this explanation in the next section.

### Language choice

Thus far, we have provided evidence consistent with a theory that both gender stereotypes and gender performance interact to produce gender inequality in interpersonal influence, but that neither gender stereotypes nor gendered performance are a dominant mechanism for that effect. We next turn to a direct evaluation of the gendered language used in the text of the conversations. Because written text is the only communication between respondents on our platform, variation in gendered performances can only be the result of different patterns of language use in the text exchanges. Furthermore, because the mislabeled partner does not know their gender has been misrepresented, changes in their language use can only arise as a reaction to the language used by their partner who has stereotyped expectations of their gender.

Complementary to the theoretical expectations of influence presented in Table 1, if gender stereotypes were the only mechanism accounting for the gender gap, we would expect to see no difference between men and women’s language use in any condition. Likewise, if gender presentation were the only mechanism accounting for the gender gap, we would expect to see gendered differences in language use that are unaffected by the mislabeling treatment. However, as the prior results suggest both mechanisms are simultaneously at work, then we expect a more nuanced result from the text analysis. Prior research has found that treating one person in a gendered conversation affects the gendered language use for both participants as a conversation progresses^55,71,72^. In this scenario, we might expect a man in the conversation to speak differently to the woman because he thinks he is talking to another man. The woman, in turn, might respond to the different tone of the conversation in kind by using more masculine language.

We use a dictionary of gendered political words developed by Roberts and Utych^73^ to examine how speech patterns differ across conditions and genders. Roberts and Utych asked both male and female human coders to evaluate the masculine or feminine connotations for each of 700 words commonly used in political conversations. The resulting scale ranges from 1.36 (for the word woman) to 6.4 (for the word man). We score the average gender connotation of the dictionary words used by each person in the political conversations. Further analyses using other natural language processing techniques reach similar conclusions (see the Supplemental Appendix).

**Figure 5:**
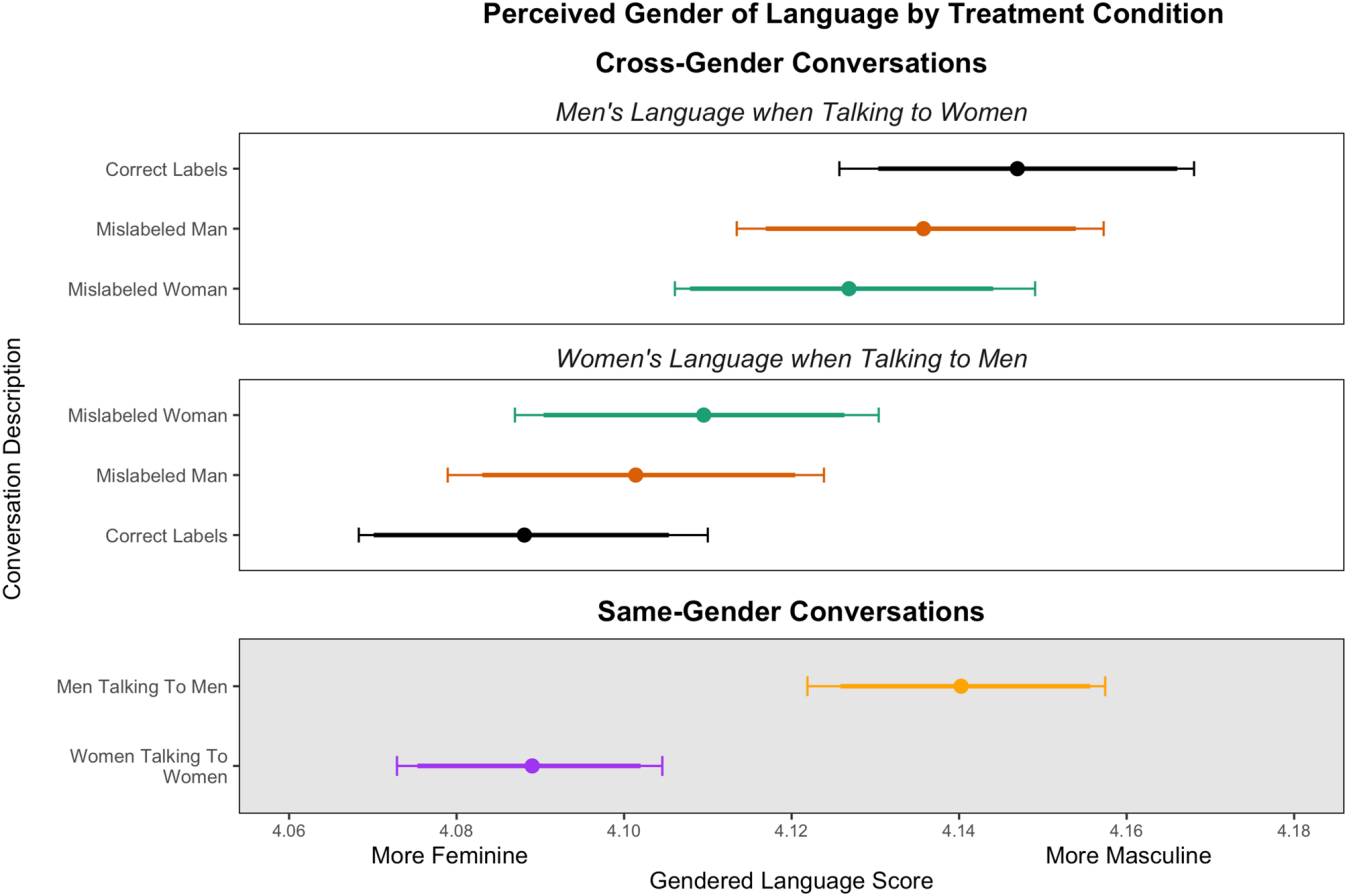
Patterns of gendered language usage in chats on the social media platform by treatment condition. X axis describes average gender connotation of words, based on the Roberts and Utych dictionary database^73^. Higher scores indicate the overall pattern of word usage is more male. Across all treatment conditions, men used more male-sounding language, and women used more female-sounding language. Mislabeling changes the average gendered language score for both the treated partner and the mislabeled partner. The “Same-Gender Conversations” panel is greyed out to emphasize the fact that comparisons involving these estimates cannot be interpreted causally.

Figure 5 shows that men in the study, particularly in the cross-gender control condition, consistently used words with more masculine connotations and women used words with more feminine connotations. This provides additional evidence that differences in gender performance, and the associated normative value attached to those differences in performance, play a role in the influence exerted by men and women in political conversations.

However, when one of the discussion partners is mislabeled, we find it changes both men’s *and* women’s behavior. For example, women use the most masculine language in situations where they themselves have been labeled as a man—*even though they do not know they have been labeled as a man*. So while there is clear evidence of gendered language use in political conversations, behavioral differences are likewise not the sole explanation for women’s lower influence in conversations. Rather, behavior appears to be dynamically impacted by the stereotyping behavior of the other person.

We believe this demonstrates the complex, emergent nature of language and gender discrimination in real life settings. The results of all four analyses show evidence that both stereotypes and behaviors are at play, and that the interaction of both produces something distinctive from either. Importantly, our findings further highlight the limitations of interventions that are non-interactive or limited to a single exchange. Gender for both participants is constructed and reinforced continually throughout the course of a dynamic interaction^74^.

## Discussion

Gender gaps in interpersonal influence are far more complex than many theories account for. Using a field experiment on an anonymous chat platform created to simulate social media conversations during the 2020 presidential primaries, we randomized people to talk with partners of different genders while being represented by avatars that were either consistent or inconsistent with their self-identified gender. This design allowed us to study how expectations of lower political competence among women (gender stereotypes) and differences in the actual text of language used by men and women (gendered performance) interact to create gender inequality in interpersonal political influence. A gender stereotype explanation would have predicted that mislabeling women as men would improve their influence in the conversation, whereas men’s influence would be lower when they were mislabeled as women. By contrast, a gendered behavior explanation would have expected no impact of mislabeling on the persuasiveness of the mislabeled individual. What we found is not consistent with either hypothesis, and instead points to the interaction of both mechanisms via role incongruity.

Using multiple metrics of influence in the conversation, we find evidence of a clear gender gap in influence in cross-gender control conversations. However, when women in the conversation are mislabeled as men, their influence does not improve, and instead may actually decrease. Likewise, mislabeling men as women reduces men’s influence and may even reverse the gender gap in influence. We use individual-level metrics of influence, accounting for the partner’s gender, and find that the gender gap seems to exist more because of the perception of the partner’s propensity to be influenced than any gender difference in the subject’s persuasiveness.

Men, in general, are much less likely to *be persuaded* than women. The good news is that men’s propensity to be persuaded is not immutable, and when women perceive their male partner to be a woman they are more effective at persuasion. Indeed, this is the only condition in which men’s attitudes significantly move as a result of conversation. However, opinions in these conversations actually diverge from one another overall, suggesting that merely changing how men are perceived and treated may not achieve consensus-oriented decision-making goals. This notable result suggests that future research should emphasize how the stereotypes about and treatment of men reinforce gender gaps as much as—or possibly even more than—stereotypes and treatment of women.

Additionally, we find gender differences in language use, and evidence that people change their language in response to both the perceived and actual gender and language use of the other person. Men and women use distinct vocabularies in political conversations, which communicate their gender to others. Additionally, men and—especially—women seem to adapt their behavior in response to the perceived gender of the person with whom they are talking. This suggests that performances of gender in political conversation are relational, constructed in response to both stereotypes attached to a conversation partner’s gender presentation and observations of their gender performance.

Thus, the complex and dynamic interpersonal construction of gender cannot be easily or durably manipulated using a single, static intervention. Proposed practical solutions and future research on women’s influence must take into account a more complex model than can be achieved by relying on just one of these explanations while holding other features constant. Allowing for this complexity in research designs is particularly important when studying conversation in online spaces, where gender cues are often more easily controlled and more often misinterpreted than in face-to-face discussion. Unfortunately, this also implies that the solutions to improving women’s influence are inherently difficult. Women cannot improve their levels of influence simply by talking more like men or “leaning in.” But neither can women become more influential without accounting for different perceptions of the persuasiveness and persuadability of both men and women.

There are several important limitations to this study. First, the discussions are limited to the realm of American politics, which is a highly gendered domain for interpersonal interaction^9,10,75^. As discussed in the introduction, gender differences in interpersonal influence also occur in many other domains. We expect that similar dynamics might be observed in other gendered contexts, but caution that the specific applicability of the results of this study to other domains should be carefully considered.

Furthermore, we only looked at Democrats. Gender in politics functions differently across parties^76,77^, and it is reasonable to expect that the effects would be different for Republican men and women than are observed among Democrats—or political parties in other countries. Nevertheless we expect Democrats to be generally more tolerant of gender differences in language, meaning these results can be interpreted as a lower bound of gender effects in the US context^34,35^. Moreover, the focus on Democrats allows us to look at the important mechanisms and experiences of intra-party persuasion and discussion, in an era when so much scholarship is looking at inter-party polarization.

Also, we considered a single, uniquely gendered primary election cycle. It is possible that our results were affected by gendered differences in candidate support and enthusiasm. In Supplemental Materials Sect. 5.2, we show that although there were gendered differences in candidate rankings in our sample—specifically, women were more likely than men to name Senator Elizabeth Warren as their top choice candidate—thermometer ratings of all candidates were quite similar. Given this similarity and our experimental randomization, we consider it unlikely that our results are driven by gendered differences in candidate support. Our sample size precludes an investigation of the interaction between respondent gender and candidate characteristics (including gender), but we consider this an important direction for future work.

Additionally, our sample is relatively White, highly educated, and excludes people who identify outside the gender binary.  When combined with the “normative” nature of these identities in unmarked situations^78^, this means that we cannot investigate possible differences in our results driven by the intersectional effects of race and class with gender or patterns for people who do not identify as women or men (see sample demographics in the Supplementary Material). Because gendered expectations and performance differ by race and class^79^, additional research is needed to further verify our results across the population. 

Finally, while we provide evidence that gender is communicated and constructed between partners using language, future research should further investigate those dynamics. Additionally, gender is performed in many ways that go beyond language. In in-person interactions, differences in vocal tone, appearance, or body language may additionally contribute to gender differences^80,81^. At the same time, the social media platform we created allowed us to experimentally manipulate gender in a series of interactions between real people. This allows us to build on the important, but limited, experimental work on gender that employs hypothetical or single-shot interactions. Indeed, these results reaffirm the need for experimental designs that attempt to fully model the complex interplay of gender performance and gender stereotypes. Additionally, proposed solutions that primarily target the attitudes or behaviors of one side of an interaction are unlikely to overcome the interdependent processes that constitute the gender gap in interpersonal influence; solutions must account for both biases and behaviors and attend to their effects on and among both genders.

## Methods

**Figure 6:**
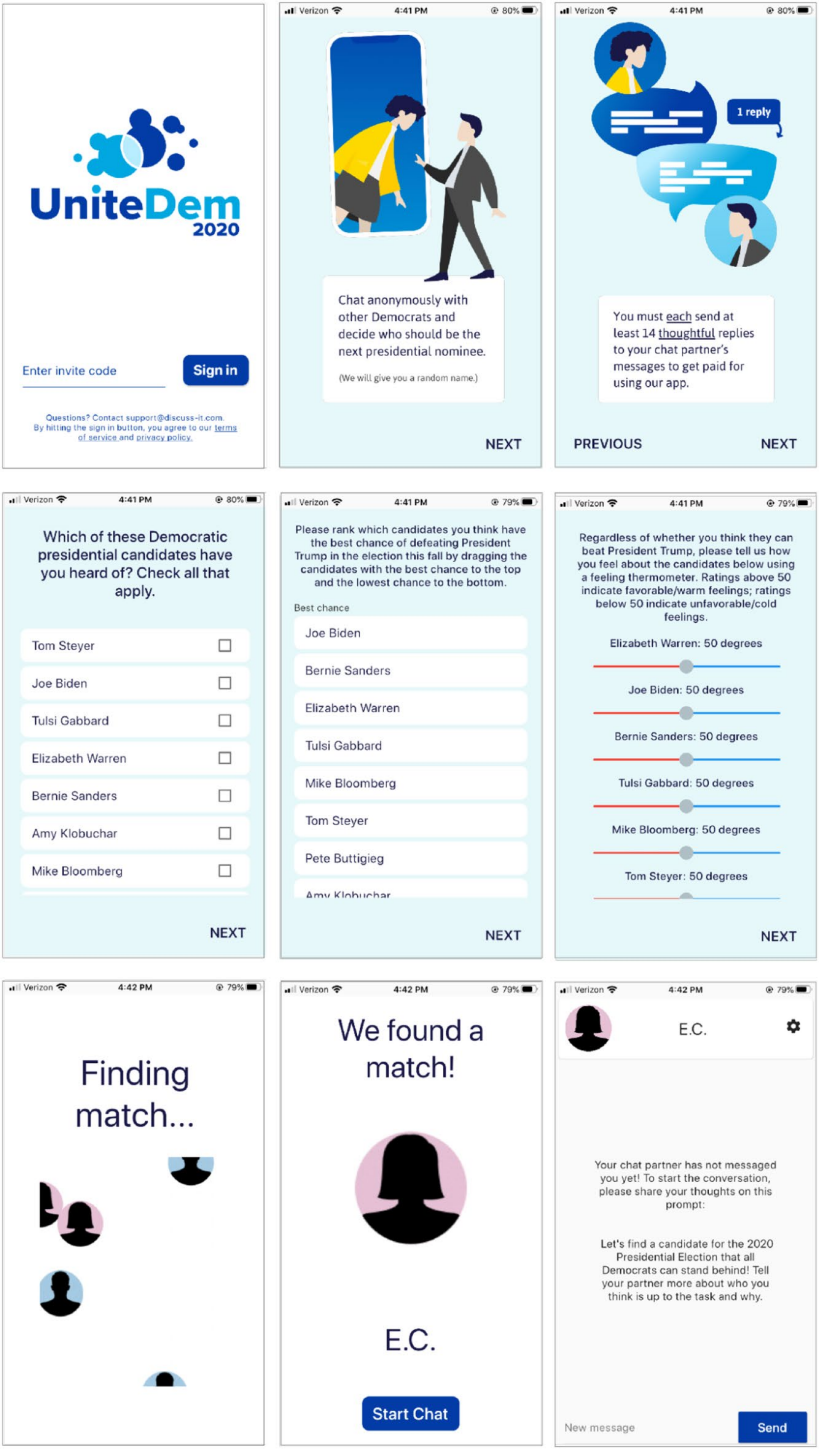
Screenshots of the Social Media Platform Used in Study. 596 Democrats were recruited to download an app. They completed a survey about their views about presidential candidates and were instructed to discuss which one was best positioned to defeat Trump in the 2020 Democratic Primary election. After completing the in-app survey, some respondents were randomly assigned an avatar (seen only by their discussion partner) that was inconsistent with their self-reported gender. After a 14 exchange chat with another Democrat in the study, respondents completed a post-survey of their attitudes.

We hired the survey firm YouGov to recruit self-identified Democrats who were told they had been randomly selected for an opportunity to earn $10 for testing a new app. Recruitment started on February 28, 2020, and the app, called UniteDem, was described as a way for Democrats to anonymously discuss which candidate was best positioned to defeat Donald Trump in the general election. Respondents were asked to install UniteDem on an iOS or Android mobile device and given an invite code that we used to assign them to one of several treatment conditions described below. Figure 6 shows the onboarding screens viewed by the user. Users were asked not to disclose any information about themselves and assigned a set of pseudonymous initials to avoid cuing gender via names. Respondents were then directed to a survey which began by asking them to select all of the candidates they were aware of prior to the study. They were then asked to rank-order these candidates in terms of their preference and assign each of them a feeling thermometer score between 0 and 100 to describe their overall opinion of the candidate, independent of their capacity to win.

Next, respondents were redirected to a screen that presented a brief video in which a series of male and female silhouettes circled around while informing the user that the app was searching for a discussion partner (see Fig. 6). The app then displayed the avatar and pseudonym initials assigned to the user’s discussion partner and redirected both users to a chat interface where the partner’s silhouette appeared on the top left corner of the screen and remained for the entire conversation. Users were not able to see the gender avatar that was assigned to them.

Conversations ended after the participants had completed 14 exchanges, where an “exchange” is defined as one user sending one message or a few successive messages followed by one or more successive messages from the other user, or at 7 pm eastern standard time on March 3, 2020 (Super Tuesday) if they had not yet completed all exchanges. All conversations where both participants completed the post survey before the Super Tuesday deadline are included in the analysis; our results are robust to excluding the eleven conversations with fewer than 14 exchanges, see Supplemental Appendix Sect. 2.1.2. The same completion standard (14 exchanges) was used in a previous experiment on a similar platform after pretesting to ensure conversations were meaningful enough and completed within a reasonable time frame^82^.

Following the conversation, the app asked respondents (N = 596; attrition is addressed in the Supplemental Appendix) to complete a post-treatment survey with the same measures of candidate rankings and ratings and indicating whether their partner influenced their opinions about the candidates (other questions are described in the Supplemental Appendix). Differences in the rankings and ratings of candidates from the pre-chat survey to the post-chat survey are used as measures of influence in the conversation, along with the subjective ratings of partner influence given by participants.

All of our research was approved by the Institutional Review Board at Duke University. Respondents provided informed consent to participate and were debriefed about the nature of the study and experimental manipulation after the conclusion of the experiment. All methods were performed in accordance with the relevant guidelines and regulations.

### Supplementary Information

Supplementary Information.

## Data Availability

Anonymized replication code and data are available at this link: https://doi.org/10.7910/DVN/37QFAX.

## Code availability

Upon publication, replication code will be made publicly available by the authors at this link: https://dataverse.harvard.edu/dataverse/chris_bail.
