# Supplementary material for: Perceived gender and political persuasion: a social media field experiment during the 2020 US Democratic presidential primary election
Source: Sci Rep. 2023 Aug 28;13:14051. doi: 10.1038/s41598-023-39359-0 (PMC10462641; doi:10.1038/s41598-023-39359-0)
Supplement: Supplementary file 1 — Supplementary Information. [file 41598_2023_39359_MOESM1_ESM.pdf]

Supplementary Materials for “Perceived Gender and Political Persuasion: A Field Experiment during the 2020 Democratic National Primary”

Aidan Combs, Graham Tierney, Fatima Alqabandi, Devin Cornell, Gabriel Varela, Andrés Castro Araújo, Lisa P. Argyle, Christopher A. Bail, Alexander Volfovsky

13 March, 2023

Contents

1 Replication Materials **1**

2 Pre-registration **1**
2.1 Deviations from the pre-registration statement . . . 1

3 Recruitment Process **3**

4 Study Recruitment Language **3**

5 Characteristics of Study Population **4**
5.1 Balance Table . . . 7
5.2 Candidate Preferences by Gender . . . 9
5.3 Sample Attrition . . . 10

6 Operationalization of Gender **13**

7 Respondent Experience on Study’s UniteDem Platform **13**

8 Manipulation Check **16**

9 Gender Reveals in Language **16**

10 Additional Text Analysis **17**
10.1 Conversation Descriptions . . . 17
10.2 Gender Differences in Language . . . 18

11 Additional Analyses **22**
11.1 Influence Gap Results . . . 23
11.2 Thermometer Rating Gap Results . . . 27
11.3 Individual-Level Influence Results . . . 28
11.4 Same Gender Conversations . . . 31

Bibliography **34**

1 Replication Materials

This document describes all materials and methods for the article "Perceived Gender and Political Persuasion: A Social Media Field Experiment during the 2020 Democratic National Primary," by Combs et al. All data, code, and the markdown file used to create this report will be available at this link on the Dataverse upon publication of the manuscript.

2 Pre-registration

The research design and hypotheses described in the main text were pre-registered using the Open Science Framework. Our pre-registration statement is available at this link.

2.1 Deviations from the pre-registration statement

Our study was a novel and complex endeavor that involved many moving parts and was conducted in a political context that was continuously shifting in unpredictable ways. As such it became clear to us that some changes to our pre-registered methods would be necessary. We summarize these changes here.

2.1.1 Differences in app design

The preregistration contains app design details and screenshots that differ in some places from that of the final app. The most important changes were tweaks to the design of our gendered avatars. The avatars deployed in the study (shown in Section 7) were colored in brighter shades of blue and pink than the avatars included in the preregistration, and the silhouettes were slightly larger. This change was made in response to the results of a pre-test that showed that versions with deeper colors prompted a stronger treatment response. In addition, we made small, non-substantive changes to the language in the settings window and discussion prompt questions after the submission of the preregistration to make them more clear, consistent, and concise in response to feedback obtained in final rounds of pre-launch app testing. Further information on the final app design is presented in Section 7.

2.1.2 Conversation completion standard

In our pre-registration statement, we write that we would tell respondents they must exchange 14 messages with their discussion partner to receive financial compensation, but counted their conversation as complete if they reached 10 exchanges. In addition, our statement does not mention that we would require everyone to have completed their conversation by Super Tuesday because our original plan was to launch the study well ahead of Super Tuesday. However, various complications related to our survey firm and last minute bugs in our mobile platform pushed our launch date back, making this new deadline necessary. Early that day, in an effort to get as many complete post-surveys as possible, we pushed all pairs who had exchanged some messages but not yet completed their conversation to our post-conversation survey. We counted their conversations as complete and included them in analysis if they completed the survey before the first polls closed at 6pm. This means that a few conversations in our final sample for conversation-level analysis (six conversations total) exchanged fewer than 10 messages. We believe this makes our results somewhat conservative, however, because these people had less time to influence or be influenced. All treatment effects that are statistically significant at least at the 5% level in our main specification and models with demographic controls remain significant when excluding the 6 conversations that had fewer than 10 exchanges or the 11 conversations that had fewer than 14 exchanges.

2.1.3 Outcome variables

In the preregistration we discuss two influence-related outcome variables: difference in partner's pre and post-conversation candidate rank and difference in partner's thermometer ratings. In the analyses presented here, we include these two measures for the respondent's pre-conversation top-choice candidate as well as a third outcome variable: partner's self-reported opinion change. We include this third measure in order to capture another more subjective, less specific dimension of opinion change. In addition, to ease interpretation

and consider the three measures simultaneously, we combine these variables to create an index measure of aggregate influence.

2.1.4 Level of analysis

Our pre-registration statement stated that we would use individuals as the level of analysis. During our research, we found it was helpful to pair this individual-level perspective with a conversation-level analysis, presented in Figures 3 and 4. This allows us to draw conclusions not only about the changes in influence for individuals, but the changes in trajectories of conversations based on their labeling conditions.

2.1.5 Modeling strategy and control variables

In our pre-registration statement, we stated we will analyze our data using linear regression and control for a number of sociodemographic variables. In our main-text analyses, we do not use these control variables, and instead directly compare effects between conditions and participant groups. As our participants were randomly assigned to conditions and our analysis of balance between conditions (Section 5) shows that our randomization succeeded in creating groups that are sociodemographically similar, including control variables was unnecessary and risked creating an overfitted model considering our relatively small sample. We also run models with these control variables in analysis reported in Section 11 and find that the results do not differ in substance or significance from results of models without control variables reported in the main text.

2.1.6 Language

Our hypotheses about gendered language differences in the pre-registration posit differences in thoughtful/cerebral and emotional language between men and women and between experimental conditions. Our text analysis described here does not examine rational and emotional language specifically, but instead investigates gender differences in use of gendered political words (in the main text) and gender differences in receptiveness, features associated with politeness, and "warm and friendly" versus "tough and firm" communication styles (supplemental materials). This is because we had not yet encountered the political dictionary created by Roberts and Utych, which is used in the main text. When we learned about it, it became clear that the clearest way to test for gendered differences in language in political conversation would be to use a method that was specifically intended to measure the gendered content of political speech. Other text analyses presented in Section 10 are likewise performed using tools we felt were well-validated and got at the spirit of the preregistered hypotheses, which is gendered differences in conversation style.

3 Recruitment Process

Between February 28th and March 6th 2020, we hired the YouGov survey firm to recruit members of its online panel who are citizens of the United States and describe themselves as Democrats (or lean Democrat if they identify as independents) to download our app. Respondents were pre-screened to ensure they owned an iOS or Android mobile device or tablet. The sample was stratified such that half of the respondents identified as male when they entered the YouGov panel, and half identified as female (see Section 6, below, for more detail on operationalization of gender). After passing a screener questionnaire to ensure they were eligible to participate in the study (using the criterion above), respondents were shown an informed consent script, in which they were offered the equivalent of $10 in YouGov's points system to participate in the study. The informed consent script explained that respondents were being recruited to test a new social media platform for Democrats called UniteDem which would require them to engage with (or reply to) another user 14 times over five days. A reply is one "turn" in a conversation— or shift between a statement between one discussion partner and the other. YouGov contacted approximately three thousand people, of whom about 1800 agreed to download the app. In total, 938 people completed the pre-survey and were assigned to a study arm. In this paper, we analyze the 596 people who completed the post survey. See Section 5 for sample characteristics.

4 Study Recruitment Language

The text we used to recruit respondents to join the study was as follows:

> You have been selected for an opportunity to receive an additional 10,000 points for testing a new app called UniteDem. UniteDem is a new chat platform where Democrats can anonymously discuss which candidate for the 2020 presidential election would best represent their party with another member of their party. The app is being used by a team of researchers to study how people make decisions about which candidates to support.
>
> To qualify for the 10,000 point bonus, you must a) install the UniteDem app on an Android or Apple smartphone (or tablet), and b) engage in a sincere conversation with another UniteDem user in which you make at least 14 replies, and answer questions about your views as well your experience on the new platform. The app will help you monitor how many replies you have made and inform you once you have reached the required threshold for payment. Although the time it takes to reply to these conversations may vary, we estimate it will take less than one hour spread across several days. You will have one week to complete the conversation.
>
> The UniteDem app employs strict privacy and data security standards. The app collects no personally identifiable information about you. Your responses will be linked only by a numeric identifier assigned to you by YouGov that includes demographic data about you and your political preferences. The data that you generate as part of this research may be shared with future researchers to ensure that any scientific findings can be replicated, and to minimize the need for additional studies.
>
> Your participation in this activity is entirely voluntary, and you have the right to decline to participate or stop participating at any point. If you choose to do this, you may request that any information obtained from you be removed by emailing the research sponsor at support@unite-dem.org.
>
> We encourage that you print or save this form for your own records.
>
> I am at least 18 years of age, and desire of my own free will to participate in this study.
>
> · Yes
>
> · No

If respondents consented to participate, they were shown the following text:

> Thank you for agreeing to install the UniteDem App. Click this link on your mobile device to be taken to the app store in order to download the app. Once installed use invite code [code specific to each respondent] to sign in. Remember that users of UniteDem are asked not to disclose any information about themselves. You must complete 14 replies with your discussion partner in order to receive your YouGov points.

5 Characteristics of Study Population

Table S1 describes the characteristics of the 1835 Democrats over age 18 who agreed to participate in our study compared to the 1201 who did not. As the table describes, those who consented were significantly younger and more interested in the news. Consenters and those who did not consent are also differently distributed throughout the country; those who consented are more likely to reside in the South and less likely to reside in the Northeast. Consenting participants were also more likely to be college graduates, have higher incomes and identify more strongly as liberals; and less likely to be white, though these differences are not statistically significant at the $p < .05$ level.

Table S1: Comparison of Consenter and Non-Consenters

	Consent N=1835	No Consent N=1201	p.overall
Age	53.4 (17.2)	58.0 (17.0)	<0.001
White: Yes	1461 (79.6%)	991 (82.5%)	0.053
College grad: Yes	1323 (72.1%)	826 (68.8%)	0.054
Income	89693 (78107)	86931 (71004)	0.344
News interest	3.60 (0.77)	3.54 (0.80)	0.038
Strength of liberal identification	0.88 (0.90)	0.86 (0.89)	0.538
Region:			0.016
Midwest	413 (22.5%)	266 (22.2%)	
Northeast	388 (21.1%)	301 (25.1%)	
South	573 (31.2%)	320 (26.7%)	
West	461 (25.1%)	313 (26.1%)	

Table S1: Characteristics of participants who consented and those who did not. Means and standard deviation reported for continuous attributes, number and percentage (in-group) reported for categorical attributes. P-values are calculated with a t-test for continuous variables and chi-square for categorical variables. The same methodology is applied in Tables S2-S5.

Of those that consented to participate in the study, 938 completed the pre-survey in its entirety. Table S2 compares those who did and did not complete that survey. These respondents were significantly younger, whiter, have more interest in the news and identify more strongly as liberals than the 897 who did not complete the pre-survey.

Table S2: Comparison of those who completed and did not complete the Pre-Survey

	Complete pre-survey N=938	Did not complete pre-survey N=897	p.overall
Age	51.2 (17.0)	55.7 (17.1)	<0.001
Gender: Women	468 (49.9%)	422 (50.8%)	0.726
White: Yes	765 (81.6%)	696 (77.6%)	0.040
College grad: Yes	692 (73.8%)	631 (70.3%)	0.113
Income	91556 (76666)	87711 (79611)	0.317
News interest	3.64 (0.75)	3.56 (0.79)	0.038
Strength of liberal identification	0.97 (0.86)	0.79 (0.93)	<0.001
Region:			0.306
Midwest	213 (22.7%)	200 (22.3%)	
Northeast	203 (21.6%)	185 (20.6%)	
South	275 (29.3%)	298 (33.2%)	
West	247 (26.3%)	214 (23.9%)	

As shown in Table S3, 746 participants sent at least 1 message to their partner. Note that this is the first time treatment (gender mislabeling and same gender partners) could affect the results, so we add that variable to the tables here. Treated means the participant is in a mislabeled *conversation*, i.e. either the participant's or their partner's gender is mislabeled. We also include the variable "No of Candidates Recognized", which is the number of Democratic Primary candidates that the participant recognized in the presurvey.

Table S3: Comparison of those who sent and did not send one message

	Did not send a message N=192	Sent at least 1 message N=746	p.overall
Age	49.7 (16.7)	51.5 (17.1)	0.176
Gender: Women	102 (53.1%)	366 (49.1%)	0.356
White: Yes	150 (78.1%)	615 (82.4%)	0.204
College grad: Yes	144 (75.0%)	548 (73.5%)	0.733
Income	97283 (87304)	90103 (73724)	0.320
News interest	3.59 (0.77)	3.65 (0.74)	0.319
Strength of liberal identification	1.01 (0.90)	0.97 (0.84)	0.531
Region:			0.164
Midwest	34 (17.7%)	179 (24.0%)	
Northeast	44 (22.9%)	159 (21.3%)	
South	66 (34.4%)	209 (28.0%)	
West	48 (25.0%)	199 (26.7%)	
No of Candidates Recognized	7.04 (1.62)	7.09 (1.48)	0.679
Treatment:			0.433
Not treated	34 (17.7%)	164 (22.0%)	
Same Gender	73 (38.0%)	269 (36.1%)	
Treated	85 (44.3%)	313 (42.0%)	

Of those that consented to use the app, completed the pre-survey and messaged their partner, only those who complete the post-survey before 5pm on March 3rd (the time at which election results were first reported on Super Tuesday) are considered in our final sample. Of the 746 respondents who messaged their partners at least once, 596 are included in our analysis.[1] These individuals tend to recognize a greater number of primary candidates when compared to respondents who did not complete the post-survey. The significant difference by treatment is further analyzed in Section 5.3.

Table S4: Comparison of those who completed and did not complete the Post-Survey

	Completed post-survey N=596	Did not complete post-survey N=150	p.overall
Age	51.3 (17.0)	52.6 (17.2)	0.384
Gender: Women	294 (49.3%)	72 (48.0%)	0.842
White: Yes	493 (82.7%)	122 (81.3%)	0.781
College grad: Yes	438 (73.5%)	110 (73.3%)	1.000
Income	90842 (74136)	87132 (72241)	0.595
News interest	3.66 (0.73)	3.62 (0.78)	0.594
Strength of liberal identification	0.99 (0.82)	0.86 (0.93)	0.114
Region:			0.700
Midwest	146 (24.5%)	33 (22.0%)	
Northeast	130 (21.8%)	29 (19.3%)	
South	162 (27.2%)	47 (31.3%)	
West	158 (26.5%)	41 (27.3%)	
No of Candidates Recognized	7.20 (1.33)	6.65 (1.91)	0.001
Treatment:			0.003
Not treated	140 (23.5%)	24 (16.0%)	

[1] Note that not all results tables in Section 11 use the full sample because the outcome cannot always be measured. Sometimes the participant may not rate a relevant candidate in both the pre- and postsurvey, which makes measuring presurvey to postsurvey change impossible.

Table S4: Comparison of those who completed and did not complete the Post-Survey *(continued)*

	Completed post-survey	Did not complete post-survey	p.overall
Same Gender	224 (37.6%)	45 (30.0%)	
Treated	232 (38.9%)	81 (54.0%)	

5.1 Balance Table

Finally, we compare the balance in our analysis sample in Table S5. The table below compares individuals in treated conversations to those in control conversations. No variables are significantly different at the 5% level across treatment and control.[2]

Table S5: Comparison of treatment and control participants

	Not treated N=140	Same Gender N=224	Treated N=232	p.overall
Age	50.9 (17.3)	52.3 (17.2)	50.5 (16.6)	0.478
Gender: Women	70 (50.0%)	108 (48.2%)	116 (50.0%)	0.915
White: Yes	119 (85.0%)	182 (81.2%)	192 (82.8%)	0.654
College grad: Yes	100 (71.4%)	164 (73.2%)	174 (75.0%)	0.746
Income	85878 (69591)	92673 (75248)	92160 (75966)	0.679
Income Not Reported	0.06 (0.25)	0.10 (0.30)	0.08 (0.27)	0.521
News interest	3.69 (0.77)	3.64 (0.77)	3.65 (0.67)	0.805
Strength of liberal identification	0.94 (0.79)	0.98 (0.81)	1.03 (0.85)	0.520
Region:				0.570
Midwest	32 (22.9%)	62 (27.7%)	52 (22.4%)	
Northeast	28 (20.0%)	49 (21.9%)	53 (22.8%)	
South	44 (31.4%)	60 (26.8%)	58 (25.0%)	
West	36 (25.7%)	53 (23.7%)	69 (29.7%)	
Political knowledge	4.71 (1.30)	4.81 (1.31)	4.70 (1.27)	0.626
No of Candidates Recognized	7.35 (1.09)	7.16 (1.41)	7.15 (1.39)	0.319

Additionally, we show that pre-conversation differences on candidate preferences do not exist for our analysis sample. These data are only collected within the UniteDem platform and high dimensional, so we only report these statistics for the final analysis sample. The three tables below show that across all treatment conditions both overall (Table S6) and separately by gender (Tables S7 and S8) we do not observe significant differences in either the top ranked candidate or candidate feeling thermometers in the pre-conversation survey. Candidates are ordered by the number of supporters in our sample.

Table S6: Pre-Conversation Candidate Preferences for All Partici-pants

	Not treated N=140	Same Gender N=224	Treated N=232	p.overall
Top Candidate (Pre-Conversation):				0.459
Joe Biden	52 (37.1%)	67 (29.9%)	77 (33.2%)	
Bernie Sanders	50 (35.7%)	64 (28.6%)	78 (33.6%)	
Elizabeth Warren	12 (8.57%)	31 (13.8%)	28 (12.1%)	

[2]Income Not Reported is included here because it is also used in covariate adjustment in Section 11. Political Knowledge is described in Section 7 and measured in the postsurvey, so it is included for the first time here.

Table S6: Pre-Conversation Candidate Preferences for All Partici-pants *(continued)*

	Not treated	Same Gender	Treated	p.overall
Mike Bloomberg	14 (10.0%)	26 (11.6%)	25 (10.8%)	
Pete Buttigieg	6 (4.29%)	15 (6.70%)	8 (3.45%)	
Amy Klobuchar	3 (2.14%)	14 (6.25%)	7 (3.02%)	
Tulsi Gabbard	2 (1.43%)	4 (1.79%)	7 (3.02%)	
Tom Steyer	1 (0.71%)	3 (1.34%)	2 (0.86%)	
Biden Thermometer	58.9 (26.9)	63.6 (26.5)	61.2 (27.4)	0.282
Sanders Thermometer	65.5 (30.9)	61.3 (29.9)	66.3 (29.4)	0.196
Warren Thermometer	66.4 (25.3)	71.4 (25.7)	72.5 (24.6)	0.077
Bloomberg Thermometer	40.9 (31.1)	42.1 (32.8)	39.9 (30.5)	0.783
Buttigieg Thermometer	61.5 (29.3)	62.5 (28.0)	58.7 (27.9)	0.367
Klobuchar Thermometer	59.2 (24.0)	60.2 (25.4)	58.4 (24.8)	0.764
Gabbard Thermometer	33.3 (30.0)	29.9 (30.2)	27.1 (29.0)	0.210
Steyer Thermometer	44.8 (25.6)	46.0 (27.7)	46.9 (26.2)	0.796

Table S7: Pre-Conversation Candidate Preferences for Women

	Not treated<br>N=70	Same Gender<br>N=108	Treated<br>N=116	p.overall
Top Candidate (Pre-Conversation):				0.621
Joe Biden	23 (32.9%)	32 (29.6%)	40 (34.5%)	
Bernie Sanders	28 (40.0%)	31 (28.7%)	36 (31.0%)	
Elizabeth Warren	7 (10.0%)	20 (18.5%)	20 (17.2%)	
Mike Bloomberg	9 (12.9%)	10 (9.26%)	13 (11.2%)	
Pete Buttigieg	2 (2.86%)	8 (7.41%)	4 (3.45%)	
Amy Klobuchar	1 (1.43%)	5 (4.63%)	2 (1.72%)	
Tulsi Gabbard	0 (0.00%)	1 (0.93%)	1 (0.86%)	
Tom Steyer	0 (0.00%)	1 (0.93%)	0 (0.00%)	
Biden Thermometer	57.3 (24.2)	61.4 (28.9)	60.9 (27.8)	0.602
Sanders Thermometer	70.0 (27.5)	61.2 (29.4)	66.7 (29.6)	0.138
Warren Thermometer	69.5 (24.6)	70.8 (26.9)	75.5 (24.3)	0.257
Bloomberg Thermometer	42.3 (32.6)	42.9 (32.4)	38.5 (32.3)	0.581
Buttigieg Thermometer	65.8 (27.5)	62.9 (26.6)	60.0 (27.8)	0.406
Klobuchar Thermometer	57.2 (22.4)	58.9 (25.2)	57.5 (24.7)	0.896
Gabbard Thermometer	29.2 (28.7)	27.4 (30.0)	20.7 (27.6)	0.177
Steyer Thermometer	48.5 (26.1)	46.2 (28.4)	47.8 (26.3)	0.859

Table S8: Pre-Conversation Candidate Preferences for Men

	Not treated<br>N=70	Same Gender<br>N=116	Treated<br>N=116	p.overall
Top Candidate (Pre-Conversation):				0.801
Joe Biden	29 (41.4%)	35 (30.2%)	37 (31.9%)	
Bernie Sanders	22 (31.4%)	33 (28.4%)	42 (36.2%)	
Elizabeth Warren	5 (7.14%)	11 (9.48%)	8 (6.90%)	
Mike Bloomberg	5 (7.14%)	16 (13.8%)	12 (10.3%)	
Pete Buttigieg	4 (5.71%)	7 (6.03%)	4 (3.45%)	

Table S8: Pre-Conversation Candidate Preferences for Men
(contin-ued)

	Not treated	Same Gender	Treated	p.overall
Amy Klobuchar	2 (2.86%)	9 (7.76%)	5 (4.31%)	
Tulsi Gabbard	2 (2.86%)	3 (2.59%)	6 (5.17%)	
Tom Steyer	1 (1.43%)	2 (1.72%)	2 (1.72%)	
Biden Thermometer	60.5 (29.5)	65.6 (24.2)	61.5 (27.1)	0.376
Sanders Thermometer	61.1 (33.6)	61.5 (30.6)	65.9 (29.3)	0.471
Warren Thermometer	63.4 (25.7)	72.0 (24.7)	69.8 (24.6)	0.077
Bloomberg Thermometer	39.4 (29.7)	41.3 (33.3)	41.3 (28.8)	0.913
Buttigieg Thermometer	57.6 (30.4)	62.1 (29.3)	57.4 (27.9)	0.432
Klobuchar Thermometer	60.9 (25.4)	61.5 (25.6)	59.3 (24.9)	0.825
Gabbard Thermometer	36.3 (30.7)	31.9 (30.4)	32.4 (29.2)	0.613
Steyer Thermometer	41.3 (24.8)	45.9 (27.2)	46.1 (26.2)	0.462

5.2 Candidate Preferences by Gender

Another key aspect of our study is participant gender, so we also report imbalances by gender on candidate preferences. Table S9 reports the same candidate preference measures split by men and women. We observe significant differences in top-ranked candidates by gender ($p < 0.01$) due to larger support for Senator Warren among women: 16% of women ranked her as their top choice while only 8% of men did the same. That imbalance is reflected in more support for Senator Klobuchar, Representative Gabbard, and Tom Steyer among men than women. However, we only see marginal differences in thermometer ratings for Senator Warren. Women rated her on average 72/100 and men 69/100. The only candidate with a significant rating difference is Representative Gabbard. Men rated her 33/100 and women 25/100, on average ($p < 0.01$). Note that these differences are not threats to causal inference because of the randomization.

Table S9: Pre-Conversation Candidate Preferences by Gender

	Men N=302	Women N=294	p.overall
Top Candidate (Pre-Conversation):			0.006
Joe Biden	101 (33.4%)	95 (32.3%)	
Bernie Sanders	97 (32.1%)	95 (32.3%)	
Elizabeth Warren	24 (7.95%)	47 (16.0%)	
Mike Bloomberg	33 (10.9%)	32 (10.9%)	
Pete Buttigieg	15 (4.97%)	14 (4.76%)	
Amy Klobuchar	16 (5.30%)	8 (2.72%)	
Tulsi Gabbard	11 (3.64%)	2 (0.68%)	
Tom Steyer	5 (1.66%)	1 (0.34%)	
Biden Thermometer	62.8 (26.6)	60.2 (27.3)	0.249
Sanders Thermometer	63.1 (30.8)	65.5 (29.1)	0.348
Warren Thermometer	69.1 (25.0)	72.3 (25.4)	0.147
Bloomberg Thermometer	40.9 (30.7)	41.0 (32.4)	0.950
Buttigieg Thermometer	59.2 (29.1)	62.5 (27.3)	0.179
Klobuchar Thermometer	60.5 (25.2)	57.9 (24.3)	0.232
Gabbard Thermometer	33.2 (30.0)	25.2 (28.9)	0.003
Steyer Thermometer	44.9 (26.3)	47.4 (27.0)	0.296

Table S10: Conversation-Level Completion Rates by Treatment Conditions.

Conversation Condition	N Incomplete	N Complete	Completion Rate
Correctly Labeled	34	70	0.673
Mislabeled Man	39	61	0.610
Mislabeled Woman	49	55	0.529
Same Gender (Men)	32	58	0.644
Same Gender (Women)	33	54	0.621

Note: N Incomplete is the number of conversations that began but at least one participant did not complete the postsurvey on time. N Complete is the number of conversations that began where both participants completed the postsurvey on time. All of these conversations are used in our main analysis. Completion Rate is the proportion of conversations in each condition that completed the postsurvey.

5.3 Sample Attrition

A particular concern here is differential attrition. If mislabeling causes participants to quit using the app, then we will be unable to measure influence in their conversation, which requires completion of the postsurvey. We focus attrition analysis on conversation-level attrition, i.e. of all conversations that began on the UniteDem platform, which ones can we use in our analysis. Individual level attrition is less relevant for our main results, Figures 3 and 4 in the main paper, which focus on conversation-level outcomes that require both conversation partners to complete the postsurvey, and harder to define. To use an individual in the analysis we need that individual's partner to complete their postsurvey because a person's influence is measured by how their partner changed.

Table S10 below shows the number of complete and incomplete conversations along with the completion rate by treatment condition. Table S11 shows logistic regression coefficients to check for significant differences in attrition by treatment either without adjusting for covariates (first column) or with adjustment by all covariates measured external to the in-app surveys by YouGov (second column). The outcome is whether the conversation was included in our analysis (both participants complete the in-app postsurvey before Super Tuesday results are released). We see that conversations where the woman was mislabeled attrited at statistically significantly higher rates than correctly labeled conversations. The odds of a conversation making it to the analysis in control are about 2:1 (67% of control conversations complete the postsurvey), the odds of a conversation making it to the analysis when the woman is mislabeled are about 1:1 (52% of conversations where the woman is mislabeled complete the post survey). The significant difference holds when adjusting for demographic covariates. Conversations where men are mislabeled as well as both same gender conversations complete at slightly lower rates than control, but the differences are not statistically significant.

One complication of this analysis is conversations involving participants who were rematched. If someone left a conversation because their partner was unresponsive, then that conversation could not enter our analysis and that outcome, whether the conversation attrited from our sample, is dependent across conversations with the same participants. To that end, Table S12 runs the same regressions as Table S11 but excludes all conversations where any partner was rematched. In this population, the attrition rate among correctly labeled conversations and conversations with mislabeled men is almost identical. Conversations with mislabeled women differentially attrit at a similar rate as in the overall sample, and the difference is statistically significant only when adjusting for covariates. Note that in Section 11, we show that our results are unchanged when excluding conversations where either partner was rematched.

Differential attrition is concerning, however, we see no differential attrition between control and conversations with mislabeled men, the group for whom we see statistically significant treatment effects. Those results that we highlight in the main paper are not affected by this issue. We do not observe significant treatment effects for mislabeling women. However, a potential explanation for that lack of effect is this attrition issue. If the conversations with mislabeled women that attrit had (unobservable to researchers) outcomes similar to other

Table S11: Attrition Analysis

	Dependent variable:	
	Completed Postsurvey	
	(1)	(2)
Man Mislabeled	−0.275 (0.293)	−0.225 (0.316)
Woman Mislabeled	−0.607* (0.287)	−0.614* (0.303)
Same Gender (M)	−0.127 (0.304)	
Same Gender (F)	−0.230 (0.304)	
Age (M)		0.005 (0.008)
White (M)		−0.051 (0.344)
College Grad. (M)		0.040 (0.298)
Income (M)		−0.001 (0.002)
Income Not Reported (M)		0.098 (0.457)
News Interest (M)		0.250 (0.244)
Political Ideology (M)		0.057 (0.146)
Northeast (M)		−0.688 (0.397)
South (M)		−1.039** (0.389)
West (M)		−0.664 (0.392)
Age (W)		0.007 (0.008)
White (W)		0.256 (0.335)
College Grad. (W)		0.118 (0.288)
Income (W)		−0.002 (0.002)
Income Not Reported (W)		−0.834 (0.473)
News Interest (W)		−0.066 (0.154)
Political Ideology (W)		0.126 (0.148)
Northeast (W)		−0.159 (0.368)
South (W)		0.333 (0.359)
West (W)		0.317 (0.361)
Constant	0.722*** (0.209)	−0.248 (1.168)
Observations	485	308
Log Likelihood	−320.832	−194.658
Akaike Inf. Crit.	651.665	435.315

Note: Each column reports logistic regression coefficients and parenthetical standard errors from regressing an indicator variable for whether the conversation could be used in our analysis (both partners completed the postsurvey) on treatment indicators and, in column two, demographic variables. Parentheticals in demographics indicate which participant they correspond to, M for man and W for woman. Note that same-gender conversations are excluded from column two because they do not have gender-identified control variables. *p<0.05; **p<0.01; ***p<0.001.

Table S12: Attrition Analysis Excluding Rematches

	Dependent variable:	
	Completed Postsurvey	
	(1)	(2)
Man Mislabeled	−0.009 (0.339)	0.046 (0.365)
Woman Mislabeled	−0.568 (0.318)	-0.725^{*} (0.340)
Same Gender (M)	0.018 (0.342)	
Same Gender (F)	−0.260 (0.329)	
Age (M)		−0.004 (0.009)
White (M)		0.386 (0.396)
College Grad. (M)		−0.395 (0.370)
Income (M)		0.004 (0.002)
Income Not Reported (M)		0.618 (0.561)
News Interest (M)		0.403 (0.264)
Political Ideology (M)		0.172 (0.173)
Northeast (M)		−0.364 (0.468)
South (M)		-1.044^{*} (0.443)
West (M)		−0.733 (0.441)
Age (W)		0.004 (0.009)
White (W)		−0.085 (0.404)
College Grad. (W)		−0.062 (0.342)
Income (W)		−0.001 (0.002)
Income Not Reported (W)		−0.806 (0.542)
News Interest (W)		−0.020 (0.182)
Political Ideology (W)		−0.036 (0.177)
Northeast (W)		−0.303 (0.438)
South (W)		0.422 (0.420)
West (W)		0.155 (0.404)
Constant	0.916^{***} (0.232)	−0.245 (1.282)
Observations	418	258
Log Likelihood	−260.483	−151.086
Akaike Inf. Crit.	530.967	348.171

Note: This table desplays the same regressions as Table S11 except it excludes all conversations where any participant was rematched. Each column reports logistic regression coefficients and parenthetical standard errors from regressing an indicator variable for whether the conversation could be used in our analysis (both partners completed the postsurvey) on treatment indicators and, in column two, demographic variables. Note that same-gender conversations are excluded from column two because they do not have gender-identified control variables. Parentheticals in demographics indicate which participant they correspond to, M for man and W for woman. $^{*}p<0.05$; $^{**}p<0.01$; $^{***}p<0.001$.

treated conversations, then our results would be stronger and potentially significant if we had observed them. Of course, if the outcomes were more similar to control, then the results would stay the same (insignificant differences). However, the latter possibility is less likely because of the differential attrition; if those who attrit in the treatment group were the same as control there should be no differential attrition.

We also note that conversations where the man is from the South attrit at higher rates than conversations where the man is from the Midwest (the baseline level for Region). However, because region is balanced across treatment conditions in expectation due to randomization and confirmed as balanced in our sample, this does not affect the estimated treatment effects.

6 Operationalization of Gender

Self-identified gender is collected by YouGov as part of their standard demographic profile questionnaires, and was not uniquely re-identified for this study. They ask "Are you. . . ? " with two gender options, "Male" and "Female." We therefore do not have any indication of the prevalence of transgender or gender nonbinary respondents in the subject pool.

7 Respondent Experience on Study's UniteDem Platform

All respondents who consented to participate in our research were sent detailed instructions about how to install the app on an iOS or Android device. Each respondent was sent an "invite code" via the survey firm and instructed to use it when they logged into the platform for the first time. The first onboarding screen on the UniteDem platform allowed people to enter their invite code and gave them information about how to contact us to address any technical issues they might have experienced during the log-in process. The second screen included a message which explained that the platform was a forum where users could chat anonymously about which candidate should represent their party in the 2020 presidential election. Users were instructed not to disclose any personal information about themselves. On the third onboarding screen, respondents were reminded they must engage in at least fourteen "exchanges" with their discussion partner to receive financial compensation. However, we considered people as treated if they complete 10 exchanges (see below). On the fourth screen, users were shown the names of all eight candidates currently vying for the presidential nomination in the Democratic Party and asked "Which of these democratic candidates have you heard of? (check all that apply)." On the fifth screen, users saw the names of the candidates they checked off in the previous screen and the following text: "Please rank which candidates you think have the best chance of defeating President Trump in the election this fall by dragging the candidates with the best chance to the top and the lowest chance to the bottom" On the sixth screen, respondents rated each candidate independent of their electability via the following question: "Regardless of whether you think they can beat President Trump, please tell us how you feel about the candidates below using a feeling thermometer. Ratings above 50 indicate favorable/warm feelings; Ratings below 50 indicate unfavorable/cold feelings."

After completing the survey portion of the app experience, users were matched with another respondent in the study. If the system was unable to match the user, the respondent was shown a screen that said "Searching for match" followed by a screen titled "No match found yet!" with the text, "We haven't found a chat partner for you yet. But don't worry! We will keep searching and notify you when we do." When a match became available, the user received a push notification on their phone and the next time they opened UniteDem, they were taken to a screen that showed the avatar of the person with whom they have been matched. The avatars constitute the study's main treatment. In the control condition and same sex conditions, both participants viewed avatars associated with the gender their partner reported when they first joined the study, while in the mislabeling treatment conditions one of the individuals was shown an avatar that did not match the identified gender of their partner. The avatars appeared to the user as part of a short .GIF video: First, blue male avatars and pink female avatars (see Figure S1) spun around and then the avatar representing their partner increased in size as the other avatars faded away, indicating to the user that their discussion partner is either female or male. Immediately afterwards, we assigned one of the following gender-neutral initials to the avatar: L.C., G.C., T.C., E.C, or B.C. In order to strengthen the treatment, the male or female avatar

icon also appears next to each message within the app's main "chat" dialogue, described in the following section.

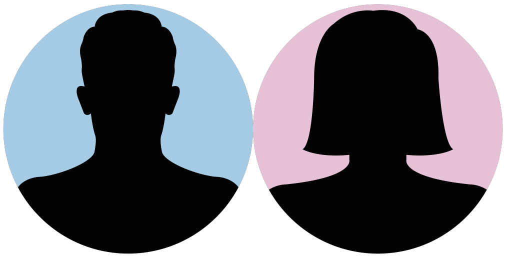

Figure S1: Avatars used to cue gender

After the "match" screen, the user was directed to the main chat interface. The first of the two users to enter the chat receives a pop-up window titled "Tell [partner initials] what you think! To begin, please tell [partner initials] what you think about the following prompt. Let's find a candidate for the 2020 Presidential Election that all Democrats can stand behind! Tell your partner more about who you think is up to the task and why." After this window closed, "Let's find a candidate for the 2020 Presidential Election that all Democrats can stand behind! Tell your partner more about who you think is up to the task and why." was shown in gray text on the background of the messenger window until one user sent a message. After closing the popup window, the user could enter their response to this question in a text window at the bottom of the screen. They were then shown the following pop-up message: "Great start! In order to receive compensation from YouGov, you'll have to reply to messages from [partner initials] at least 14 times." Below this message they were shown the following text "Your chat partner has not messaged you yet! Feel free to close UniteDem while you wait. We will send you a notification when they do!" The user received a pop-up notification on their phone when their discussion partner replied to their first message. If they tapped on this message, they were redirected to the study's main chat window.

The second user to visit the chat interface received a popup window that read "To begin, we asked [partner initials] to share their stance on this prompt: 'Let's find a candidate for the 2020 Presidential Election that all Democrats can stand behind! Tell your partner more about who you think is up to the task and why.' Write back to complete your first interaction on UniteDem!" After the user sent a message, they were also shown a popup window titled "Great start!" with text "In order to receive compensation from YouGov, you'll have to reply to messages from [partner initials] at least 14 times." When the users reached 2 exchanges with each other, they received a third pop up notification titled "Chat Rating" where they are asked to give a "thumbs up" or a "thumbs down" response to their discussion partner's last message. This popup read "You can tell us whether you like what your partner says throughout the conversation. How would you rate [partner initials]'s last message?"

Once users reached the app's chat interface, they had access to a settings page via a gear icon at the top right of the page. The settings page gave them the option to report or block their partner, contact the UniteDem team (staffed by us), see how many exchanges they had completed with their partner, and view chat prompts. The chat prompts window was available via a button titled "Conversation stuck?" which showed the following message: "Think about some questions you could ask [partner initials]. For example: What is most important to you when choosing a candidate? Which candidate do you think has the best chance of beating Donald Trump in November? Which candidate do you think has the best ideas about climate change? Health care? Foreign policy? Inequality?" These prompts were meant to serve as conversation starters and were optional for participants to use.

If users had not completed 14 replies with their discussion partner within approximately one week of starting or by the weekend prior to Super Tuesday (whichever came first), the app sent a notification to the last respondent who did not reply to their conversation partner as follows: "We noticed you haven't replied to [partner initials] in a while, and the deadline is coming up! If you don't respond soon, we'll have to assign

them a new partner. Need ideas to keep the conversation going? Visit the settings menu for suggestions." Meanwhile, the other chat partner received the following message "We've noticed [partner initials] hasn't responded to you in a while and the deadline is coming up. Don't worry, we sent them a reminder. We will assign you a new partner if they don't respond soon." Prior to the weekend before Super Tuesday, people whose partners remained unresponsive were automatically rematched to others whose partners had also become unresponsive. This rematching was done in a way that preserved the experimental conditions. People were still assigned opposite gender partners in their same experimental condition and retained their same label. In this case, respondents were asked to reply to the new discussion partner's messages the same number of times that would have been required of the member of the pair who had previously completed the most replies. The results we report in the main text of our manuscript hold regardless of whether people who were re-matched (56 respondents) are dropped from the sample (see Section 11). Reminders were still sent to people who became unresponsive or whose partners became unresponsive during the last few days of the study period, but the rematching language was removed because these people would not have had sufficient time to complete a conversation with a new partner before the cutoff date of Super Tuesday.

Once respondents completed 14 replies to each other, or on the morning of Super Tuesday if they had exchanged at least one message at that time, they were redirected to an exit survey within the app. In addition to asking respondents to re-rank their preferred candidates and complete a thermometer rating of each candidate, we asked them how much they agree or disagree with the following statements on a seven point scale: 1) "[partner's initials] influenced my views on the candidates for the 2020 Democratic primary election"; 2) "I influenced [partner's initials] views on the candidates for the 2020 Democratic primary election"; 3) [partner's initials] was open to hearing my views on who is the best candidate for the 2020 Democratic primary election"; 4) [partner's initials] was well-informed and knowledgeable about the candidates for the 2020 Democratic primary election. Next, respondents were asked, "Which of these options do you think best describes your partner's gender? [Man, Woman, Not Sure]." If respondents answered "not sure" they were asked the following question: "Please make your best guess about your partner's gender" [Man, Woman].

Next, we asked each app user to complete a series of questions designed to measure their perceived and actual knowledge of politics. To measure perceived political knowledge, we asked: "How much do you think you know about politics compared to most Americans" with the following response options: "Much more than most Americans," "A little more than most Americans," "A little less than most Americans," and "Much less than most Americans." We asked a battery of six questions to gauge actual political knowledge: 1) "What job or office is now held by John Roberts? [Chair of the Democratic National Committee, Senate Majority Leader, Chief Justice of the Supreme Court, Chair of the Republican National Committee, Don't know]; 2) Who is the current Speaker of the U.S. House of Representatives? [Nancy Pelosi, Paul Ryan, Marco Rubio, Don't know]; 3) How long is the term of office for a U.S. Senator? [2 years, 4 years, 6 years, 8 years, Don't know]; 4) Which party holds a majority of seats in the U.S. House of Representatives? [Democrats, Republicans, Don't know]; 5) Taking your best guess, what percentage of the U.S. Congress do you think are women? [5%, 24%, 46%, 61%, Don't know]; 6) Of the nine members currently serving on the U.S. Supreme Court, how many are women? [1, 2, 3, 4, 5, Don't know]. The political knowledge variable used in our analysis is the number of the above questions that were correctly answered, treating skipped questions as incorrect.

Then, respondents were asked about their overall interest in politics with the following questions: 1) "Some people seem to follow what's going on in government and public affairs most of the time, whether there's an election going on or not. Others aren't that interested. Would you say that you follow what's going on in government and public affairs... [Most of the time, some of the time, only now and then, hardly at all]."

Finally, we asked two additional questions designed to capture political engagement: "1) Do you plan to donate money to any of the candidates? If so, please select the ones you plan to support; 2) Do you plan to volunteer for any of the candidates? If so, please select the ones you plan to volunteer for.

For descriptions on the conversations themselves, see Section 10.1.

8 Manipulation Check

The results indicate our manipulation of the perceived gender of their chat partner was largely successful. Participants talking to a opposite gender, correctly-labeled partner correctly guessed that their partner's gender was the same as the avatar they saw 89% of the time; participants talking to an opposite gender, incorrectly-labeled partner incorrectly guessed that their partner's gender was the same as the avatar they saw 64% of the time. 56% of males talking to females labeled as male guessed that their partner was male, and 72% of females talking to males labeled as female guessed that their partner was female. This check suggests the manipulation worked in the majority of cases, and also previews differences in gendered behavior in the conversations. We provide additional analyses below that suggest focusing on the sub-group of respondents who were successfully manipulated does not alter the findings reported in the main text of our article.

9 Gender Reveals in Language

Even though respondents were instructed not to reveal information about themselves during their chats, we could not prevent them from doing so, either explicitly or implicitly. Such gender "reveals" could bias our analysis if the gender being discussed is incongruous with the alter's icon because they could reveal the nature of the treatment in the mislabeled case. As a result, members of our research team read through every chat and manually coded instances when a participant "revealed" their gender to their discussion partner.

In the sample of conversations used for the analysis, only 14 chats contained mentions of a participant's gender, with some chats containing multiple mentions. These do not include gender reveals that took place after our outcome is measured (i.e. after Super Tuesday). The most common type of "reveal"—use of a gendered term for a significant other—is still somewhat ambiguous, as it could be taken to provide information on either gender or sexuality, depending on context and strength of the respondent's normative assumptions of hetereosexuality.

We report a detailed list of the 23 mentions:

- "my husband": 10
- "my wife": 6
- "as a mother": 1
- "may offer a perspective from a democratic woman": 1
- "i tried not to get pregnant": 1
- "as a white woman": 1
- "as woman": 1
- "being a 60 yo white male": 1
- "as an old white woman": 1

Of the 14 respondents who revealed their gender through the text, 12 were correctly labeled. Only two women labeled as men mentioned or implied being women to their chat partners. The exact counts for gender reveals by individual labeling conditions and gender are listed below.

- Women labeled as women: 6
- Men labeled as men: 6
- Women labeled as men: 2
- Men labeled as women: 0

To assess possible bias related to gender reveals through language, we re-ran our models after dropping those that produced the language described above. The results of this analysis did not significantly change the findings reported in the main text of our manuscript.

10 Additional Text Analysis

10.1 Conversation Descriptions

In this section we provide some high-level summary statistics of the conversations themselves. An “exchange” occurs when one participant sends a message and then their partner replies without any word count requirement. Consequently, we treat a “message” as the concatenation of all text sent by a single respondent without a partner reply, essentially combining all consecutive text sent by a single person. The average message was 43 words long (IQR of 17 to 55 words) and the median time between messages was about 1.5 minutes (IQR of 1 to 15 minutes). The mean number of messages in conversation was 31 and the median was 30. Note that for each participant to have 14 exchanges, at least 28 messages must be sent in the conversation.

As a heuristic measure of conversation substance, we include a word cloud created from the word counts across all conversations. Figure S2 shows the 200 most common words scaled and colored by their overall frequency.[3] Trump was the most-used word, reflecting general concern with the upcoming general election across many conversations. Senator Sanders and now-President Biden were also frequently mentioned.

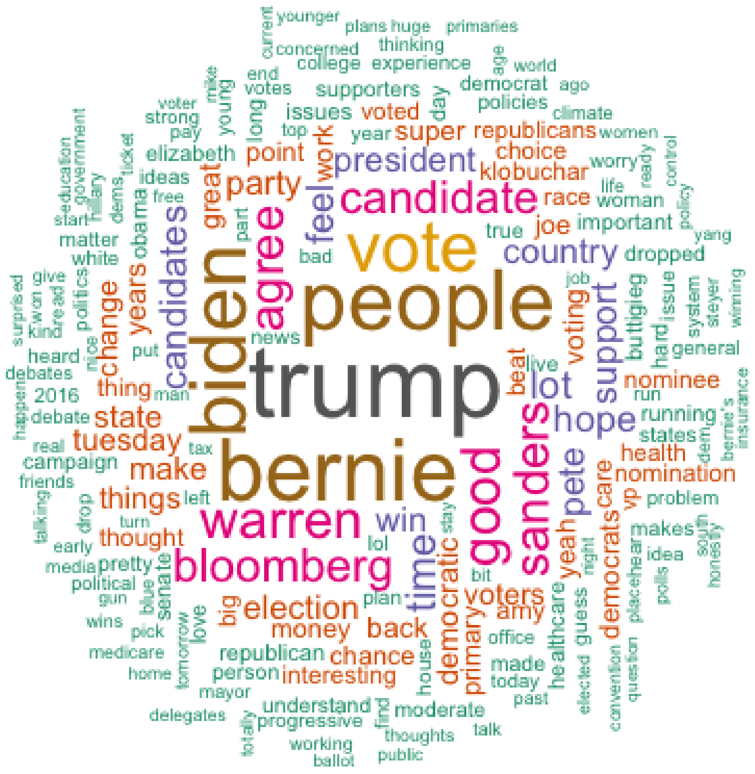

Figure S2: Most Frequent Words in Conversations

[3] Stopwords from the SMART lexicon were removed.

10.2 Gender Differences in Language

This section details the text analyses we conducted in order to understand gender differences in language. First, we describe the technique developed by Roberts and Utych (2020) that we leverage in the main text of our manuscript. This technique employs a dictionary of 700 words with corresponding ratings that measure how "feminine" or "masculine" a word sounds. The ratings vary from 1.36 (for the word woman) to 6.40 (for the word man). It was developed with the explicit purpose of understanding the nexus between gender, language, and politics. As part of their findings, Roberts and Utych show that dominant and negatively valenced words—as measured by the Affective Norms for English Words (ANEW) database—are more likely to be rated as masculine. Overall, the authors suggest that individual gendered words have a subtle influence over candidate support. The dictionary was created using Amazon Turk. As Roberts and Utych describe:

> *We recruited participants from Amazon's Mechanical Turk (MTurk) in June 2018 to rate words on their perceptions of masculinity or femininity. A total of 175 participants were recruited for the study, with each participant rating a randomly assigned hundred words, of the seven hundred total that were rated. Due to random assignment, words were rated by a minimum of fifteen raters, and a maximum of forty-four, with a mean of 25 (SD = 5.19). Participants were paid $1 for completing this task, which took an average of six minutes to complete.*
>
> Roberts and Utych (2020, 43)

This dictionary-based approach is not without limitations. Most importantly, our corpus contains 9,156 unique words (counting nouns, verbs, and adjectives), which means that using this dictionary leads to a significant loss of information. The words contained in this dictionary amount, on average, to 4% of the total vocabulary used within each chat. Furthermore, eleven of the chats did not use any of these unique words. Figure S3 conveys this loss of information in the form of a histogram.

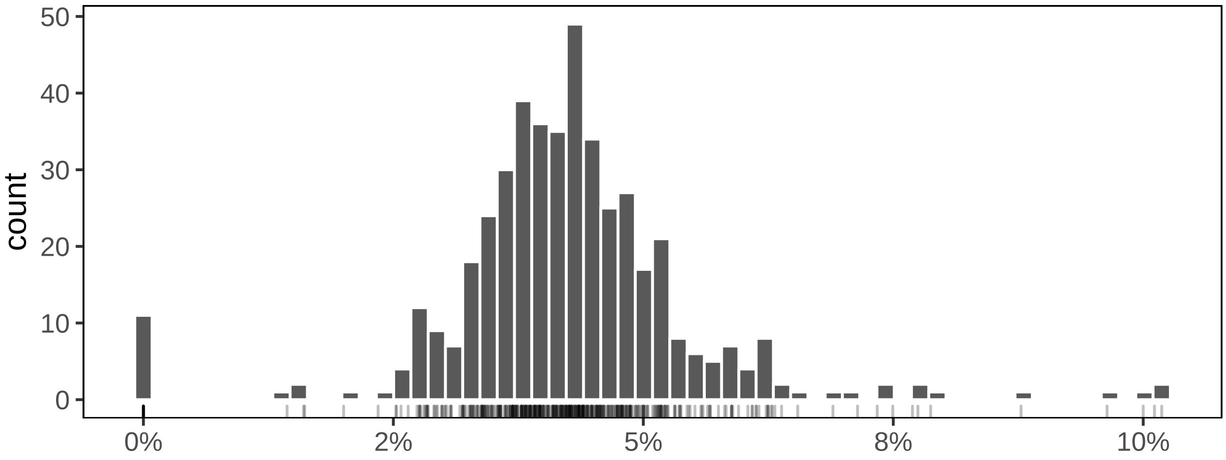

Figure S3: Percentage of words from the Roberts & Utych (2020) dictionary that are used within each chat. The words in this dictionary represent (on average) approximately 4% of all words used in each chat.

Another notable feature of this dictionary is that, on average, male raters tend to rate words as sounding more "masculine," whereas women rate words as sounding more "feminine." Figure S4 reproduces the result in the main article but separating the averages by the gender of the rater. This does not change the results, but it does highlight the fact that the measurement we relied upon is noisy.

To complement the results revealed from using Roberts and Utych's dictionary, we employed the "conversational receptiveness" classifier developed by Yeomans et al. (2020) to measure the extent to which participants use language in a way that communicates "one's willingness to thoughtfully engage with opposing views." Their method focuses on the stylistic properties of text, rather than its substance. Messages judged to be receptive often include: positive statements (e.g., "that's great"); explicit acknowledgement (e.g., "I understand," "I see your point"); points of agreement (e.g., "I agree," "you're right"); and hedging or indicators of uncertainty

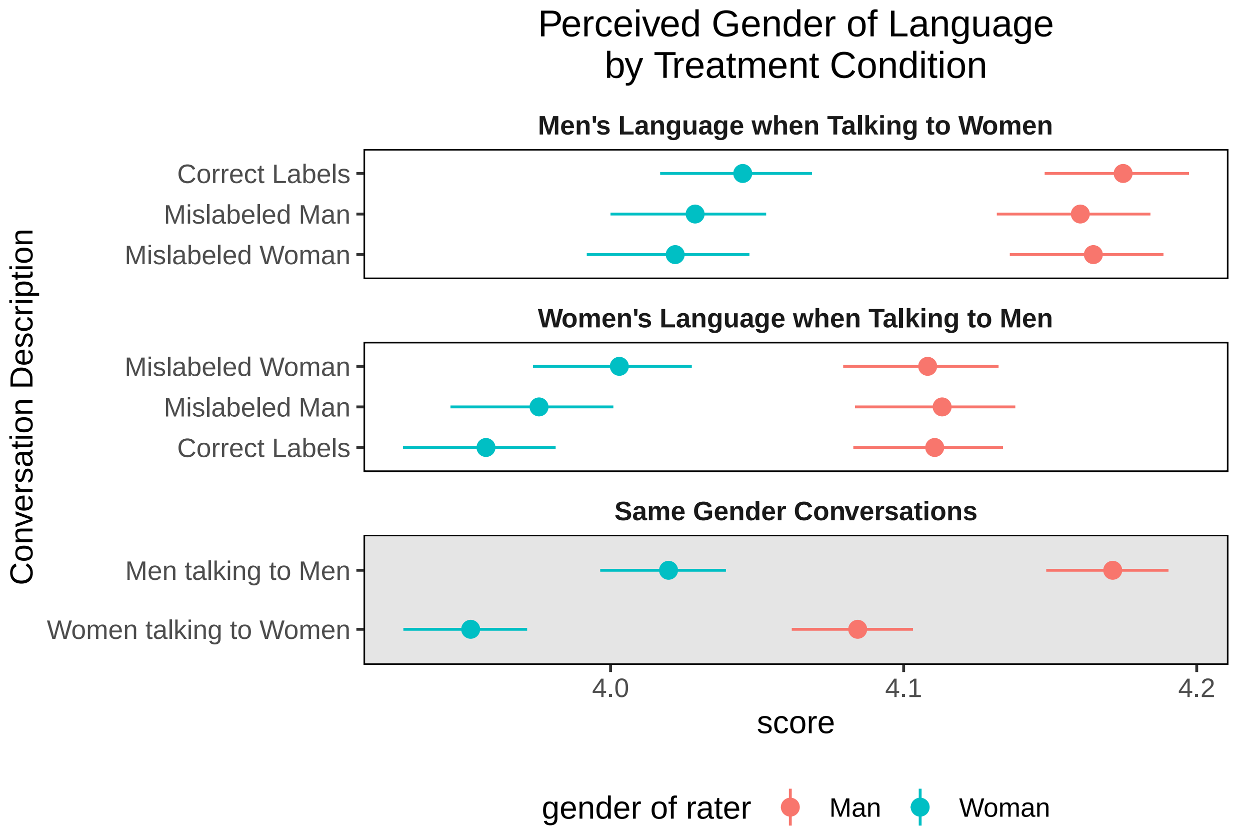

Figure S4: Patterns of gendered language usage in chats on the social media platform by treatment condition. X axis describes average gender connotation of words, based on the Roberts and Utych dictionary database. Higher scores indicate the overall pattern of word usage is more male. Across all treatment conditions, men used more male-sounding language, and women used more female-sounding language. The results remain substantively the same when accounting for the gender of the rater in the Roberts and Utyc dictionary.

used to soften claims. Finally, they note those whose language includes markers of receptiveness are rated more persuasive by others, a metric similar to the outcome of our experiment. The classifier predicts "conversational receptiveness" on the basis of a set of features related to polite speech.

Figure S5 suggests that women tend to be more receptive when talking to correctly labeled men, and less receptive when they are mislabeled (i.e., when their conversation partner thinks they are talking to another man). In other words, women tend to be less receptive when their male counterparts talk to them as if they were "one of the guys." Men on the other hand, tend to be less receptive when they are engaged in conversation with correctly labeled women—though the differences are minimal.

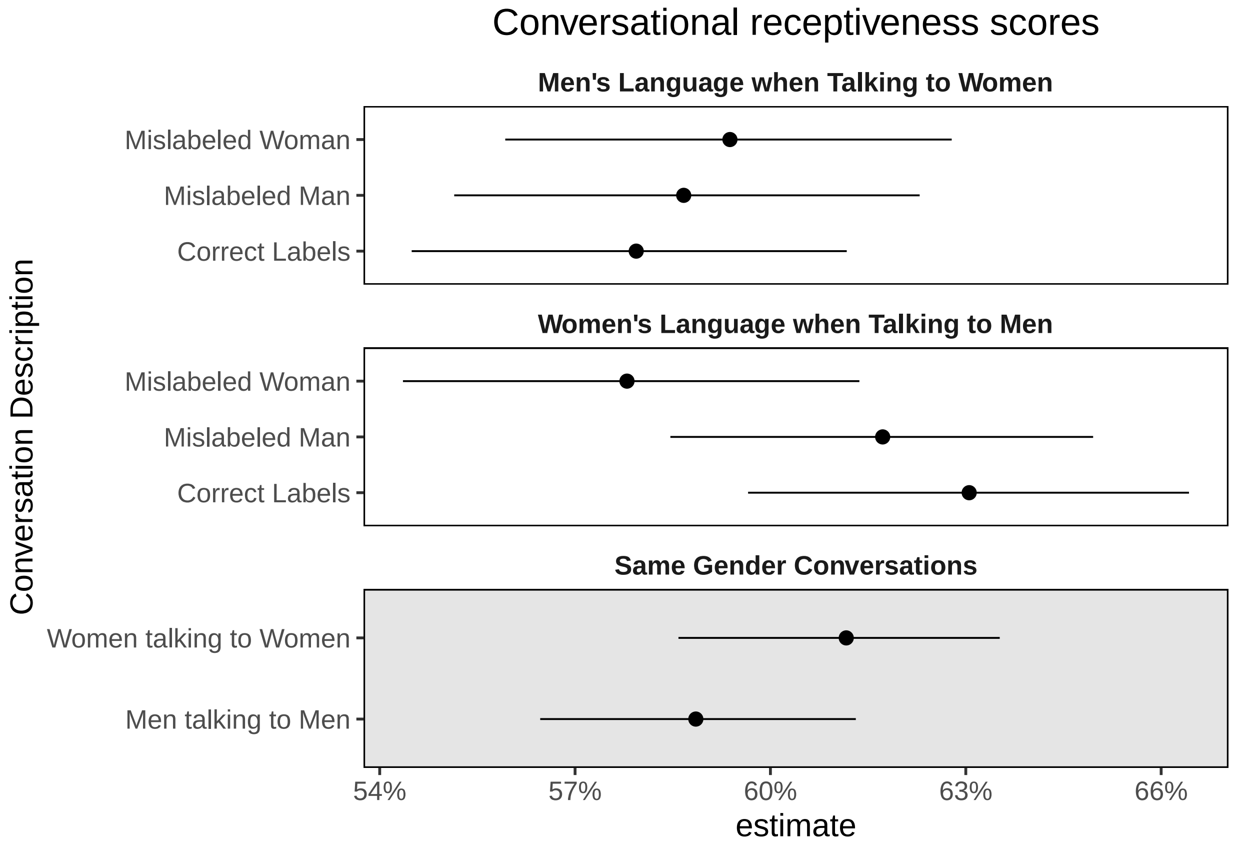

Figure S5: The receptiveness scores are presented as the probabilities that each individual user is "receptive to opposing views" according to the classifier developed in Yeomans et al. (2020). The lines represent 90%confidence intervals. Both the classifier and the code that turns text data into politeness features are available as part of the "politeness" package in R (Yeomans, Kantor, and Tingley 2018).

We also examined whether there are gender differences in the likelihood that a message uses a "warm and friendly" communication style as opposed to a "tough and firm" communication style. This classifier is trained on a different dataset collected by Yeomans, Kantor, and Tingley (2018) using Amazon Mechanical Turk and uses the same set of stylistic features related to polite speech described above. The training dataset was collected using Amazon Mechanical Turk, half of the workers were told to use a "warm and friendly" communication style in a message involving an online economic transaction; the other half were told to use a "tough and firm" communication style. Figure S6 suggests that men tend to be more "warm and friendly" towards mislabeled women and more "tough and firm" towards correctly labeled women. Women also tend to be more "warm and friendly" towards mislabeled men but "tough and firm" towards mislabeled women.

Finally, we examine which polite speech features are predictive of whether the participant is a man or a woman. To do this, we used a standard feature selection algorithm to identify the stylistic features that might be predictive of gender. The resulting coefficients are estimated to maximize the predictive accuracy of

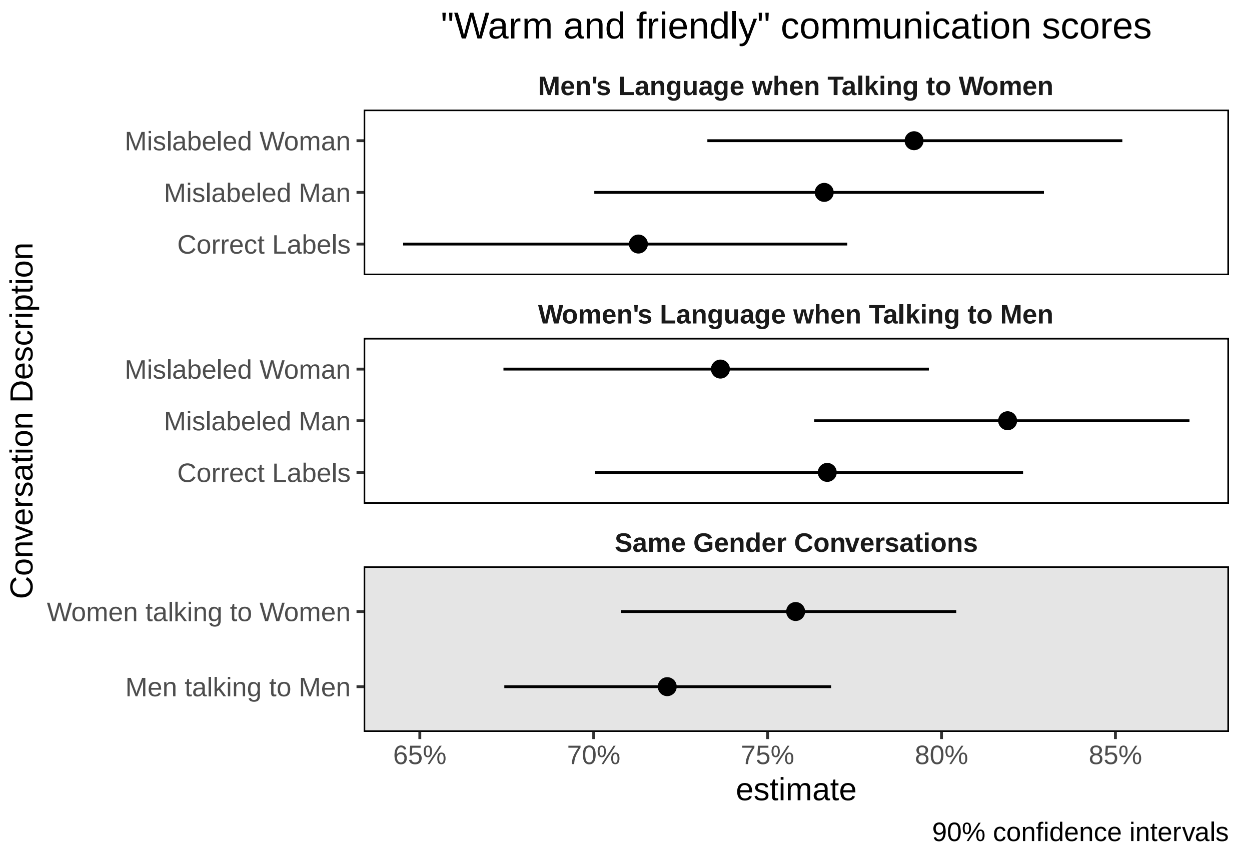

Figure S6: The warm and friendly communication scores are presented as the probabilities that each individual user communicating using a "warm and friendly" style. The lines represent 90% confidence intervals. This classifier uses the previously mentioned politeness features and a training dataset that is also included in the "politeness" package.

the model as a whole, which means that we must caution against interpreting them as "true" coefficients, much less to be causal in any way.

The most notable aspect of this analysis is that it is very hard to predict switching based on these features (the in-sample accuracy is 61%), which means the differences are very subtle. Figure S7 compares messages written by all participants. The features most predictive of men are the expression "By the way" and use of swear words, whereas the features most predictive of women are direct requests ("Can you") and expressions of gratitude ("thank you," "I appreciate," etc.).

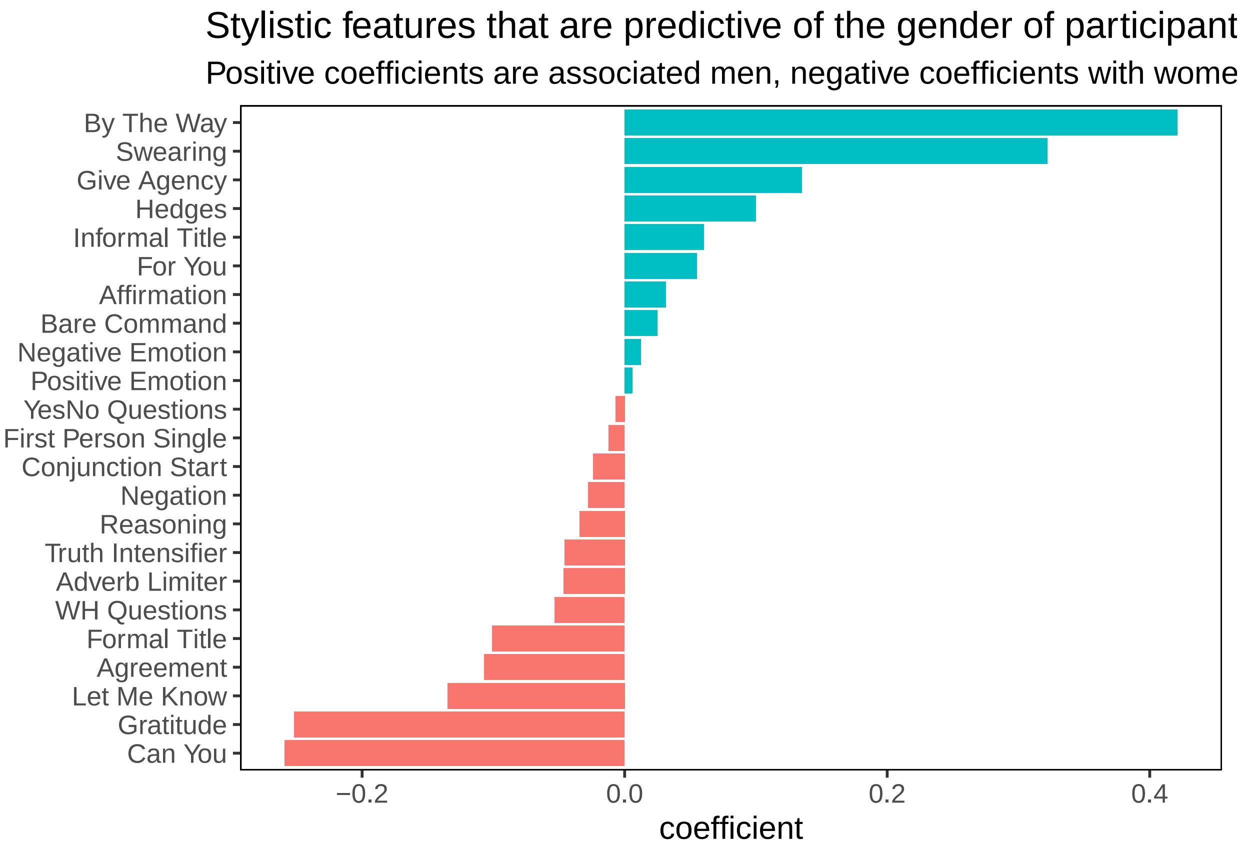

Figure S7: Coefficients of logistic regression with LASSO and cross-validation to predict whether the participant was male using glmnet R package (Friedman, Hastie, and Tibshirani 2010).

11 Additional Analyses

This section details several additional analyses and robustness checks for results reported in Figures 3-5 of the main text of our manuscript. We report regression results for the following: main paper results, adding demographic control variables, dropping conversations where either participant was rematched due to a non-responsive partner, and dropping conversations where participants saw through the gender mislabeling. The final two are not properly causal because they require conditioning on a post-treatment variable, so the results should be interpreted with caution.

The final column in main paper Figure S5 reports results on same gender conversations. Because of complexities in interpretation, we address these conversations separately in Section 11.4. Other results presented here apply only to cross-gender conversations.

The regression model is specified as follows. Y_i is the outcome for conversation i, MM_i is an indicator variable that is 1 if the man is mislabeled and 0 otherwise, WM_i is an indicator variable that is 1 if the woman is

mislabeled and 0 otherwise. X_i is a vector of control variables for both individuals in conversation i. We estimate the model below with OLS regression:

$$Y_i = {}_0 + {}_1 MM_i + {}_2 WM_i + \gamma^T X_i + \epsilon_i, \epsilon_i \sim N(0, \sigma^2)$$

When the outcome is individual, as in main paper Figure S5, the same equation form is used by treatment indicators are for whether the individual is mislabeled (1 for yes, 0 for no) and whether the individual's partner is mislabeled (1 for yes, 0 for no). In the case of cross-gender conversations, models are only estimated on individuals of the same actual gender so there is no dependence or interference across units in the same regression. For example, women only talk to men, so by analyzing individual-level results among only women, we preserve independence across observations. The final column in main paper Figure S5 reports results on same gender conversations. Due to the difficulties detailed in Section 11.4, those results are reported without demographic controls and with standard errors clustered to reflect conversation-level dependence.

To ease interpretation of baseline values, we exclude covariates X_i from results reported in the main text. We show that the results do not change in magnitude or significance when including them. Moreover, dropping conversations where someone was rematched also generally increases the size and significance of treatment effects. Note that when the outcome variable is influence measured on partner rankings or thermometers the regressions have a smaller sample sizes because that influence measure (the partner's pre-post change in feelings or rankings of someone's top candidate in the presurvey) cannot be computed if someone does not rank or rate their partner's top candidate in the presurvey. The survey measure of influence is available for all participants.

11.1 Influence Gap Results

This section reports results in Tables S13-S16 focusing on the influence gap metric presented in Figure 3 of the main paper. Table S13 recreates the results presented in the main paper. Next, Table S14 adds demographic covariates as controls. `Age` is the individuals age in years. `White` is a dummy variable where 1 indicates the individual is white, 0 indicates otherwise. `College Grad.` is a dummy variable where 1 indicates the individual graduated from college, 0 indicates otherwise. `News Interest` is a 5-point scale indicating how much interest the individual has in following the news. `Political ideology` is a 5-point scale indicated how liberal or conservative the individual is, positive values indicate more liberal ideology. This is the same variable as `Strength of liberal identification` in Section 5. `Northeast`, `South`, and `West` are dummy variables indicating what region of the country the individual lives in (`Midwest` is the omitted category). `Income` is income in 1000s of dollars. `Income Not Reported` is an indicator variable for whether the participant elected to not disclose their income to YouGov. `N. Cand. Recognized` is the number of 2020 Democratic Primary candidates the individual recognized in the in-app presurvey. `Political Knowledge` is the number of correctly answered political questions in the in-app survey described in Section 7. The gender of the relevant participant is indicated in parentheses; M for men and W for women. Finally, Tables S15 and S16 report the main results (without demographic controls) for subpopulations of only conversations where neither individual was rematched (Table S15) or conversations where if someone was mislabeled, their partner was deceived by the mislabeling (Table S16). The success of the deception was measured by whether the partner of the mislabeled person guessed the gender of their partner's avatar, i.e. they guessed their partner's actual gender incorrectly. Our results are the same for each of these sub populations.

All significant results presented in Figure 3 in the main text of our manuscript (Table S13 here) stay significant under these robustness checks. In conversations where the man is mislabeled, the influence gap is significantly smaller (the woman has more relative influence) than in correctly-labeled conversations on the influence index and the partner-reported survey measure. We also see significant effects when women are mislabeled indicating that mislabeling women increases the influence gap on select individual measures (the woman has less relative influence) than in correctly-labeled conversations when subsetting the treated conversations to those where both partners believed the gender of the avatars corresponded to their partner's gender on select influence measures but not the aggregate index. This is again consistent with the results that we report in the main text, a woman's influence is not improved when labeling her as a man.

Table S13: Treatment Effects on Influence Gap (Main Paper Results)

	Influence Gap Measured with:			
	Index	Partner Survey	Rankings	Thermometers
	(1)	(2)	(3)	(4)
Mislabeled Man	−0.657** (0.247)	−0.575* (0.242)	−0.457 (0.232)	−0.297 (0.227)
Mislabeled Woman	0.321 (0.254)	0.290 (0.249)	0.274 (0.239)	0.345 (0.234)
Constant	0.355* (0.168)	0.319 (0.165)	0.133 (0.158)	0.238 (0.154)
Observations	150	186	150	150
R^2	0.091	0.061	0.058	0.046
Adjusted R^2	0.078	0.051	0.045	0.033
Residual Std. Error	1.266	1.384	1.192	1.165
F Statistic	7.333***	5.959**	4.509*	3.524*

*p<0.05; **p<0.01; ***p<0.001

Table S14: Treatment Effects on Influence Gap (with Demographic Controls)

	Influence Gap Measured with:			
	Index	Partner Survey	Rankings	Thermometers
	(1)	(2)	(3)	(4)
Mislabeled Man	−0.666* (0.259)	−0.572* (0.242)	−0.391 (0.241)	−0.348 (0.251)
Mislabeled Woman	0.384 (0.261)	0.323 (0.245)	0.469 (0.243)	0.306 (0.254)
Age (M)	0.004 (0.006)	−0.009 (0.006)	0.005 (0.006)	0.013* (0.006)
White (M)	−0.353 (0.291)	−0.074 (0.277)	−0.138 (0.271)	−0.403 (0.283)
College Grad. (M)	0.048 (0.264)	0.081 (0.247)	−0.333 (0.246)	0.187 (0.257)
News Interest (M)	0.184 (0.264)	0.232 (0.241)	0.384 (0.246)	−0.129 (0.257)
Political Ideology (M)	0.085 (0.131)	0.161 (0.127)	0.018 (0.122)	−0.025 (0.128)
Northeast (M)	−0.360 (0.312)	−0.154 (0.298)	−0.467 (0.290)	0.087 (0.303)
South (M)	−0.620* (0.307)	−0.187 (0.293)	−0.260 (0.286)	−0.450 (0.299)
West (M)	−0.427 (0.300)	0.007 (0.282)	−0.462 (0.280)	−0.225 (0.292)
Income (M)	−0.001 (0.001)	0.001 (0.001)	−0.002 (0.001)	−0.001 (0.001)
Income Not Reported (M)	−0.115 (0.396)	−0.029 (0.372)	0.034 (0.369)	−0.277 (0.385)
N. Cand. Recognized (M)	0.005 (0.121)	−0.023 (0.095)	−0.030 (0.112)	0.191 (0.117)
Pol. Knowledge (M)	0.206 (0.114)	0.265* (0.107)	0.057 (0.106)	0.026 (0.111)
Age (W)	0.009 (0.007)	0.016* (0.007)	0.007 (0.007)	−0.004 (0.007)
White (W)	−0.326 (0.325)	−0.656* (0.295)	−0.206 (0.303)	0.111 (0.316)
College Grad. (W)	0.190 (0.243)	0.138 (0.226)	0.261 (0.226)	−0.008 (0.236)
News Interest (W)	−0.395** (0.148)	−0.316* (0.136)	−0.308* (0.138)	−0.098 (0.144)
Political Ideology (W)	0.155 (0.127)	0.227 (0.124)	0.149 (0.119)	0.033 (0.124)
Northeast (W)	0.176 (0.331)	−0.100 (0.315)	0.174 (0.308)	0.232 (0.322)
South (W)	0.196 (0.303)	−0.125 (0.285)	0.586* (0.282)	−0.065 (0.295)
West (W)	−0.128 (0.307)	−0.517 (0.287)	0.328 (0.286)	−0.005 (0.299)
Income (W)	−0.003 (0.002)	−0.002 (0.002)	−0.003 (0.002)	−0.001 (0.002)
Income Not Reported (W)	0.355 (0.463)	−0.113 (0.467)	0.860* (0.431)	0.037 (0.450)
N. Cand. Recognized (W)	0.008 (0.106)	−0.036 (0.085)	0.127 (0.099)	−0.042 (0.103)
Pol. Knowledge (W)	−0.116 (0.094)	−0.171 (0.088)	−0.069 (0.087)	−0.022 (0.091)
Constant	0.502 (1.381)	0.474 (1.192)	−1.109 (1.285)	−0.115 (1.342)
Observations	150	186	150	150
R^2	0.289	0.287	0.279	0.166
Adjusted R^2	0.138	0.171	0.127	−0.010
Residual Std. Error	1.224	1.293	1.140	1.190
F Statistic	1.918**	2.467***	1.831*	0.944

Note: Regression coefficients for results presented in the main text Figure 3 with demographic controls added. Parentheticals in demographics indicate which participant they correspond to. *p<0.05; **p<0.01; ***p<0.001.

Table S15: Treatment Effects on Influence Gap (No Rematching Subpopluation)

	Influence Gap Measured with:			
	Index	Partner Survey	Rankings	Thermometers
	(1)	(2)	(3)	(4)
Mislabeled Man	−0.743** (0.257)	−0.696** (0.247)	−0.417 (0.246)	−0.335 (0.238)
Mislabeled Woman	0.396 (0.265)	0.357 (0.255)	0.349 (0.254)	0.381 (0.246)
Constant	0.315 (0.174)	0.343* (0.169)	0.065 (0.166)	0.176 (0.161)
Observations	138	173	138	138
R^2	0.118	0.090	0.059	0.055
Adjusted R^2	0.104	0.080	0.045	0.041
Residual Std. Error	1.267	1.364	1.212	1.174
F Statistic	8.992***	8.449***	4.264*	3.935*

Note: Regression coefficients for results presented in the main text Figure 3 while excluding all conversations where either participant was rematched. *p<0.05; **p<0.01; ***p<0.001.

Table S16: Treatment Effects on Influence Gap (Believed Avatar Subpopluation)

	Influence Gap Measured with:			
	Index	Partner Survey	Rankings	Thermometers
	(1)	(2)	(3)	(4)
Mislabeled Man	−0.629* (0.275)	−0.707** (0.261)	−0.447 (0.260)	−0.229 (0.248)
Mislabeled Woman	0.636 (0.321)	0.613* (0.294)	0.324 (0.303)	0.763** (0.289)
Constant	0.296 (0.172)	0.268 (0.163)	0.124 (0.162)	0.175 (0.155)
Observations	102	127	102	102
R^2	0.122	0.125	0.057	0.097
Adjusted R^2	0.104	0.111	0.038	0.078
Residual Std. Error	1.213	1.274	1.146	1.094
F Statistic	6.867**	8.843***	3.004	5.301**

Note: Regression coefficients for results presented in the main text Figure 3 for only conversations where the individuals believed the displayed avatar corresponded to their partner's gender. *p<0.05; **p<0.01; ***p<0.001.

11.2 Thermometer Rating Gap Results

This section analyzes the same robustness checks for main paper Figure 4. Table S17 presents all robustness checks. Our results are unchanged under all alternative specifications e xcept f or t he B elieved Avatar subpopulation, where the effects are only significant at the 10% level.

Table S17: Treatment Effects on Termometer Rating Gaps

	Robustness Chceks for Thermometer Rating Gaps:			
	Main Result	Demographic Controls	No Rematches	Believed Avatar
	(1)	(2)	(3)	(4)
Mislabeled Man	4.186* (1.725)	4.537* (1.761)	4.957** (1.748)	3.610 (1.932)
Mislabeled Woman	2.175 (1.775)	1.416 (1.780)	2.149 (1.801)	2.573 (2.177)
Age (M)		0.035 (0.045)		
White (M)		−1.837 (2.009)		
College Grad. (M)		2.884 (1.796)		
News Interest (M)		−0.098 (1.752)		
Political Ideology (M)		0.555 (0.925)		
Northeast (M)		−2.095 (2.159)		
South (M)		−2.922 (2.126)		
West (M)		−0.969 (2.056)		
Income (M)		0.006 (0.010)		
Income Not Reported (M)		−2.813 (2.705)		
N. Cand. Recognized (M)		−0.573 (0.687)		
Pol. Knowledge (M)		0.080 (0.781)		
Age (W)		0.015 (0.052)		
White (W)		−3.700 (2.160)		
College Grad. (W)		−2.618 (1.643)		
News Interest (W)		−0.333 (1.030)		
Political Ideology (W)		−1.768 (0.902)		
Northeast (W)		5.490* (2.292)		
South (W)		2.671 (2.082)		
West (W)		1.625 (2.093)		
Income (W)		−0.016 (0.012)		
Income Not Reported (W)		5.516 (3.393)		
N. Cand. Recognized (W)		−0.841 (0.617)		
Pol. Knowledge (W)		1.168 (0.642)		
Constant	−1.888 (1.182)	7.290 (8.721)	−2.463* (1.199)	−2.476* (1.213)
Observations	185	185	172	126
R^2	0.031	0.232	0.046	0.030
Adjusted R^2	0.021	0.106	0.034	0.014
Residual Std. Error	9.818	9.382	9.595	9.394
F Statistic	2.952	1.836*	4.030*	1.905

Note: Regression coefficients for results presented in the main text Figure 4. Column 1 reports main text results. Column 2 adds demographic controls. Column 3 excludes all conversations where any participant was rematched. Column 4 includes only conversations where the individuals believed the displayed avatar corresponded to their partner's gender. *p<0.05; **p<0.01; ***p<0.001.

11.3 Individual-Level Influence Results

This section reports results in Tables S18-S21 focusing on cross-gender conversation individual-level influence presented in Figure 5 of the main paper. Table S18 presents results used in main text Figure 5. Note that the effects of partner mislabeling correspond to the opposite-gender panel in Figure 5, e.g. the effect of mislabeling the man on the woman's influence is measured in the regression using only women to compare women with mislabeled, male partners to women with correctly-labeled, male partners. After adding the same demographic controls as in the above robustness check, the same treatment effect remains significant: mislabeling men significantly influences their partner's influence. Table S20 reproduces the main results after excluding conversations with rematches, and we observe the same significant effects as reported in the main text along with an additional significant effect that mislabeling men decreases their influence. Table S21 includes only individuals who believed the avatar represented their partner's gender. The significance of results is the same as those reported in the main text.

We perform analyses inclusive of the same gender conversations in the following section, Section 11.4, because of additional interpretational and statistical issues in the analysis highlighted in that section. Due to those issues, we report the individual level results in regressions both without (this section) and with those additional observations (next section). Our conclusions are consistent across the analyses.

Table 18: Treatment Effects on Individual Influence (Main Paper Results)

	Influence Index Measured Among:	
	Men	Women
	(1)	(2)
Self Mislabeled	−0.339 (0.187)	−0.188 (0.142)
Partner Mislabeled	0.187 (0.194)	0.330* (0.140)
Constant	0.520*** (0.128)	0.147 (0.095)
Observations	161	172
R^2	0.044	0.070
Adjusted R^2	0.032	0.059
Residual Std. Error	0.997	0.765
F Statistic	3.629*	6.354**

Note: Regression coefficients for results presented in the main text Figure 5. *p<0.05; **p<0.01; ***p<0.001.

Table S19: Treatment Effects on Individual Influence (with Demographic Controls)

	Influence Index Measured Among:	
	Men	Women
	(1)	(2)
Self Mislabeled	−0.369 (0.197)	−0.183 (0.147)
Partner Mislabeled	0.232 (0.202)	0.326* (0.147)
Age (Self)	0.002 (0.005)	−0.004 (0.004)
White (Self)	−0.127 (0.229)	0.094 (0.177)
College Grad. (Self)	0.138 (0.205)	−0.214 (0.136)
News Interest (Self)	−0.036 (0.203)	0.124 (0.084)
Political Ideology (Self)	0.163 (0.102)	−0.047 (0.076)
Northeast (Self)	−0.021 (0.242)	0.120 (0.190)
South (Self)	−0.351 (0.240)	−0.004 (0.172)
West (Self)	−0.240 (0.235)	0.395* (0.173)
Income (Self)	−0.001 (0.001)	0.0004 (0.001)
Income Not Reported (Self)	−0.044 (0.305)	0.039 (0.280)
N. Cand. Recognized (Self)	−0.012 (0.077)	0.049 (0.053)
Pol. Knowledge (Self)	0.084 (0.088)	−0.026 (0.053)
Age (Partner)	0.006 (0.006)	0.001 (0.004)
White (Partner)	−0.228 (0.255)	0.202 (0.167)
College Grad. (Partner)	0.041 (0.186)	0.064 (0.148)
News Interest (Partner)	−0.198 (0.116)	−0.349* (0.145)
Political Ideology (Partner)	0.108 (0.098)	0.030 (0.077)
Northeast (Partner)	0.248 (0.256)	0.046 (0.180)
South (Partner)	0.124 (0.237)	0.156 (0.176)
West (Partner)	0.255 (0.237)	0.037 (0.170)
Income (Partner)	−0.002 (0.001)	0.001 (0.001)
Income Not Reported (Partner)	0.395 (0.359)	0.089 (0.225)
N. Cand. Recognized (Partner)	0.009 (0.078)	−0.057 (0.070)
Pol. Knowledge (Partner)	−0.117 (0.073)	−0.076 (0.065)
Constant	1.209 (0.992)	1.341 (0.775)
Observations	161	172
R^2	0.221	0.223
Adjusted R^2	0.069	0.084
Residual Std. Error	0.977	0.755
F Statistic	1.459	1.604*

Note: Regression coefficients for results presented in the main text Figure 5 with demographic controls added. Parentheticals in demographics indicate which participant they correspond to. *p<0.05; **p<0.01; ***p<0.001.

Table S20: Treatment Effects on Individual Influence (No Rematching Subpopluation)

	Influence Index Measured Among:	
	Men	Women
	(1)	(2)
Self Mislabeled	−0.408* (0.198)	−0.213 (0.147)
Partner Mislabeled	0.234 (0.206)	0.343* (0.144)
Constant	0.517*** (0.136)	0.174 (0.097)
Observations	148	160
R^2	0.062	0.080
Adjusted R^2	0.049	0.068
Residual Std. Error	1.015	0.761
F Statistic	4.778**	6.791**

Note: Regression coefficients for results presented in the main text Figure 5 while excluding all conversations where either participant was rematched. *p<0.05; **p<0.01; ***p<0.001.

Table S21: Treatment Effects on Individual Influence (Believed Avatar Subpopluation)

	Influence Index Measured Among:	
	Men	Women
	(1)	(2)
Self Mislabeled	−0.303 (0.191)	−0.204 (0.163)
Partner Mislabeled	0.417 (0.231)	0.343* (0.166)
Constant	0.524*** (0.127)	0.164 (0.105)
Observations	129	139
R^2	0.067	0.065
Adjusted R^2	0.053	0.051
Residual Std. Error	0.965	0.804
F Statistic	4.547*	4.738*

Note: Regression coefficients for results presented in the main text Figure 5 for only conversations where the individuals believed the displayed avatar corresponded to their partner's gender. *p<0.05; **p<0.01; ***p<0.001.

11.4 Same Gender Conversations

This section reports in Tables S22-S25 an analysis of the same gender conversations reported in Figure 5. These regressions hold constant the gender of the target of influence, preserving the propensity to be influenced across comparisons. Thus, the conversations between two men are added to the regressions analyzing women's influence on men, and the conversations between two women are added to the regressions analyzing men's influence on women. Contrasts between same-gender and any opposite-gender conversations should not be interpreted as identifying causal effects *of gender* because only conversation pairs were randomly assigned, while participant's actual gender is clearly not randomized. As such, the contrast between womens' influence on men and mens' influence on men does not identify the causal effect of one's own gender on influence over men. Men and women differ on many observed and unobserved characteristics. Thus, we cannot say that observed differences in influence are causally attributable to gender. We can say that influence on someone was higher or lower because that someone's partner was a man as opposed to a woman, but we cannot attribute that difference to the gender difference. The difference in influence could be caused by some unobserved characteristic that varies with gender. In addition, by including both participants from the conversation in the same regression, the observations are now not independent. Consequently, we report cluster-robust standard errors with clustering at the conversation level.

Table S22: Treatment Effects on Individual Influence (Main Paper Results)

	Influence Index Measured Among:	
	Influence on Men	Influence on Women
	(1)	(2)
Self Mislabeled	−0.188 (0.147)	−0.339 (0.180)
Partner Mislabeled	0.330* (0.134)	0.187 (0.196)
Same Gender	−0.002 (0.144)	−0.077 (0.153)
Constant	0.147 (0.092)	0.520*** (0.113)
Observations	281	257
R^2	0.032	0.025
Adjusted R^2	0.021	0.014
Residual Std. Error	0.914	1.053
F Statistic	3.040*	2.177

*p<0.05; **p<0.01; ***p<0.001

We observe that influence over men and influence over women does not differ by the gender of the person doing the influencing regardless of whether demographic controls are added. Note that the results for self mislabeling and partner mislabeling in Table S22 have identical point estimates to the results in Table S18. However, adding the demographic control variables produces different point estimates because the "Self" control variables are not balanced across genders. Those control variables have different distributions across the same gender and opposite gender conversations.

The only differences from the main text results are increases in statistical significance for the influence of mislabeled men on women. After either adjusting for demographic controls (Table S23) or excluding rematches (Table S24), mislabeled men have significantly less influence over correctly labeled women than correctly labeled men do.

Table S23: Treatment Effects on Individual Influence (with Demographic Controls)

	Influence Index Measured Among:	
	Participants Infuencing Men	Participants Infuencing Women
	(1)	(2)
Self Mislabeled	−0.182 (0.152)	−0.381* (0.180)
Partner Mislabeled	0.293* (0.141)	0.231 (0.199)
Same Gender	−0.079 (0.157)	−0.039 (0.165)
Age (Self)	−0.001 (0.004)	0.001 (0.004)
White (Self)	−0.033 (0.138)	−0.159 (0.152)
College Grad. (Self)	−0.087 (0.106)	0.042 (0.165)
News Interest (Self)	0.149* (0.071)	0.066 (0.141)
Political Ideology (Self)	0.029 (0.070)	0.193* (0.093)
Northeast (Self)	−0.016 (0.148)	0.057 (0.205)
South (Self)	−0.010 (0.158)	−0.330 (0.184)
West (Self)	0.250 (0.156)	−0.178 (0.201)
Income (Self)	0.0001 (0.001)	−0.001 (0.001)
Income Not Reported (Self)	0.125 (0.238)	0.108 (0.240)
N. Cand. Recognized (Self)	0.033 (0.043)	0.040 (0.055)
Pol. Knowledge (Self)	−0.050 (0.046)	0.060 (0.076)
Age (Partner)	0.005 (0.004)	0.005 (0.004)
White (Partner)	0.030 (0.151)	−0.175 (0.188)
College Grad. (Partner)	−0.012 (0.139)	0.074 (0.142)
News Interest (Partner)	−0.287** (0.088)	−0.262* (0.111)
Political Ideology (Partner)	0.051 (0.075)	−0.043 (0.081)
Northeast (Partner)	−0.127 (0.149)	0.142 (0.192)
South (Partner)	−0.104 (0.185)	−0.082 (0.183)
West (Partner)	−0.068 (0.152)	0.107 (0.210)
Income (Partner)	0.001 (0.001)	−0.002 (0.001)
Income Not Reported (Partner)	−0.028 (0.179)	0.215 (0.227)
N. Cand. Recognized (Partner)	−0.044 (0.051)	0.003 (0.064)
Pol. Knowledge (Partner)	−0.102 (0.061)	−0.100 (0.060)
Constant	1.270 (0.649)	1.029 (0.720)
Observations	281	257
R^2	0.141	0.214
Adjusted R^2	0.050	0.121
Residual Std. Error	0.900	0.994
F Statistic	1.543*	2.305***

Note: Regression coefficients for results presented in the main text Figure 5 with demographic controls added. Parentheticals in demographics indicate which participant they correspond to. Cluster-robust standard errors are reported with clustering at the conversation level to account for dependence between participants who conversed with each other. *p<0.05; **p<0.01; ***p<0.001.

Table S24: Treatment Effects on Individual Influence (No Rematching Subpopluation)

	Influence Index Measured Among:	
	Influence on Men	Influence on Women
	(1)	(2)
Self Mislabeled	−0.213 (0.154)	−0.408* (0.190)
Partner Mislabeled	0.343* (0.136)	0.234 (0.211)
Same Gender	−0.026 (0.149)	−0.074 (0.159)
Constant	0.174 (0.095)	0.517*** (0.121)
Observations	265	244
R^2	0.036	0.035
Adjusted R^2	0.025	0.023
Residual Std. Error	0.918	1.066
F Statistic	3.238*	2.887*

Note: Regression coefficients for results presented in the main text Figure 5 while excluding all conversations where either participant was rematched. Cluster-robust standard errors are reported with clustering at the conversation level to account for dependence between participants who conversed with each other. *p<0.05; **p<0.01; ***p<0.001.

Table S25: Treatment Effects on Individual Influence (Believed Avatar Subpopluation)

	Influence Index Measured Among:	
	Influence on Men	Influence on Women
	(1)	(2)
Self Mislabeled	−0.204 (0.170)	−0.303 (0.173)
Partner Mislabeled	0.343* (0.158)	0.417 (0.277)
Same Gender	0.023 (0.149)	−0.020 (0.165)
Constant	0.164 (0.099)	0.524*** (0.117)
Observations	232	213
R^2	0.030	0.036
Adjusted R^2	0.017	0.022
Residual Std. Error	0.934	1.044
F Statistic	2.342	2.592

Note: Regression coefficients for results presented in the main text Figure 5 for only conversations where the individuals believed the displayed avatar corresponded to their partner's gender. Cluster-robust standard errors are reported with clustering at the conversation level to account for dependence between participants who conversed with each other. *p<0.05; **p<0.01; ***p<0.001.

Bibliography

Friedman, Jerome, Trevor Hastie, and Rob Tibshirani. 2010. "Regularization Paths for Generalized Linear Models via Coordinate Descent." *Journal of Statistical Software* 33 (1): 1.

Roberts, Damon C., and Stephen M. Utych. 2020. "Linking Gender, Language, and Partisanship: Developing a Database of Masculine and Feminine Words." *Political Research Quarterly* 73 (1): 4050.

Yeomans, Michael, Alejandro Kantor, and Dustin Tingley. 2018. "The Politeness Package: Detecting Politeness in Natural Language." *The R Journal* 10 (2): 489–502. https://journal.r-project.org/archive/2018/RJ-2018-079/index.html.

Yeomans, Michael, Julia Minson, Hanne Collins, Frances Chen, and Francesca Gino. 2020. "Conversational Receptiveness: Improving Engagement with Opposing Views." *Organizational Behavior and Human Decision Processes* 160: 131148.
